# New System for Simultaneous Measurement of Oxygen Consumption and Changes in Wine Color

**DOI:** 10.3390/molecules29010231

**Published:** 2023-12-31

**Authors:** Marioli Carrasco-Quiroz, Ana María Martínez-Gil, Ignacio Nevares, Maria del Alamo-Sanza

**Affiliations:** 1 Department of Analytical Chemistry, UVaMOX—Universidad de Valladolid, 34004 Palencia, Spain; mariolialejandra.carrasco@estudiantes.uva.es (M.C.-Q.); anamaria.martinez.gil@uva.es (A.M.M.-G.); 2 Department of Agroforestry Engineering, UVaMOX—Universidad de Valladolid, 34004 Palencia, Spain

**Keywords:** spectral scanning, wines, oxidation, sensor, oxygen kinetics, spectral fingerprint

## Abstract

The design, construction and validation of a device for the accurate measurement of the dissolved oxygen content in wine and simultaneously the variation of its spectral fingerprint is presented. The novelty of this system is due to two innovative approaches. First, robustness in measurements is obtained by using cuvettes designed to simultaneously measure the dissolved oxygen and color. Secondly, automatic monitoring is performed to ensure that measurements are always taken at the same cuvette position. The fine-tuning of the device with the study of white and red wines makes it possible, on the one hand, to establish the appropriate measurement conditions and, on the other hand, to determine the amount of oxygen required to cause specific changes in the wine spectrum, information that could not be obtained until now. The preliminary results are very interesting, presenting precise data on the amount of oxygen consumed by the wine and the variations in its visible spectrum, thus reflecting the modification of the responsible phenolic compounds. This information is of great interest, since it helps to optimize the handling of the wine and, if necessary, to moderate the uptake of oxygen in each type of wine to ensure the maintenance of the color during the winemaking and conservation processes of each type of wine. The results of the experiments indicate that this new instrument is feasible and accurate for detecting oxygen changes during wine production.

## 1. Introduction

The oxygen present in wines affects the antioxidant compounds of wine, mainly the phenols, compounds carrying out chemical transformations that affect the color, structure, body, and astringency of wines [1,2,3]. Changes in the color of wine, one of the most important attributes, can occur due to several factors; one of the most important is the exposure to oxygen [4,5,6]. The most appropriate and most used technique to quantify the color change of wines is visible spectroscopy measurements, a fast, economical, and easy-to-use technique that allows different compounds to be determined with high sensitivity, although with low specificity. Currently, the use of luminescent systems is widespread because it is possible to measure dissolved oxygen without altering the sample, an aspect that facilitates the monitoring of oxygen consumption in wine. The published papers on the effect of oxygen consumption in wines describe the characteristics before and after the process, due to the impossibility of measuring the characteristics of the wine without altering the process under study [7,8,9]. Therefore, these studies do not present results on the effect of different known amounts of oxygen on the evolution of the wine [8,9].

**Figure 1:**
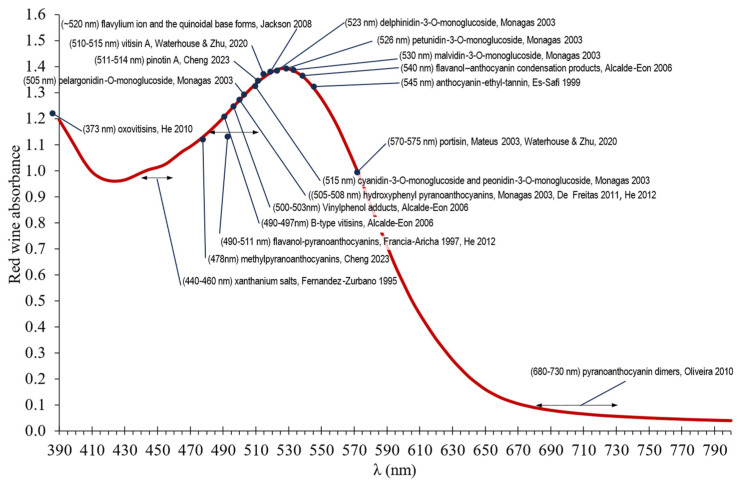
Most significant wavelengths and associated compounds on the wine spectrum [14,15,16,17,18,21,22,23,24,29,30,34,35,36].

Contact with oxygen in white wines is usually detrimental due to low antioxidant compounds, affecting to aroma, color, and flavor and reflected in the loss of fruity aroma, the presence of aromas associated with oxidation, and the appearance of golden-brownish colors due to browning [10,11,12,13]. Compounds present in white wine, such as metal ions, organic acids, and phenolic compounds, are susceptible to oxidation and can therefore cause browning. Browning tests have shown the importance of the content of these components together with the level of SO_2_ and pH [11]. The main phenolic compounds in a white wine that has not had prolonged contact with the skins or been in contact with wood are hydroxycinnamic acids. These compounds do not appear to play a direct role in the browning of white wine, because there is little correlation between their concentrations and susceptibility to browning [12]. However, there is a good correlation between flavonols and browning sensitivity, especially with (+)-catechin and (−)-epicatechin [12,14], with hydroxycinnamic acids playing a role in oxidation when coupled to flavanols. The golden color of white wine, with a wavelength increase between 440 and 460 nm, is produced by mechanisms related to the oxidation of phenolic molecules, the polymerization of flavonoids, and the oxidation of tartaric acid. Figure 1 shows the most significant wavelengths and associated compounds on the wine spectrum [15,16,17,18,19,20,21,22,23,24,25,26,27,28,29,30,31,32,33,34,35,36].

In red wines, the main source of color is anthocyanins or their derivatives. The free anthocyanins tend to decrease since they are very unstable [37,38]. The color of monomeric anthocyanins depends on the B-Ring substituents—the more hydroxyl groups, the bluer the color, and the more methoxyl groups, the redder the color—maintaining the absorption maximum in the visible spectrum between 505 and 530 nm (Figure 1). Monomeric anthocyanins are predominantly found in a dynamic equilibrium between five main molecular forms, binding to the bisulfite (colorless), the flavilium cation (red color), the quinoidal base (blue color), the hemiketal pseudobase or carbinol (colorless), and the cis and trans chalcone (yellow-orange colors), with the pH of the wine disposed towards the hemiacetal state, i.e., colorless. However, the wine can maintain its red color due to reactions and associations involving more complex molecules, with the formation of these depending on the other molecules reacting with them and other factors, with oxygen being one of the most important factors. The absorbance spectrum in the visible range that determines the color depends directly on the molecular structure and more specifically on the degree of delocalization of electrons within a given molecule [39]. Thus, color is divided into three categories: color due to co-pigmented anthocyanins, free pigments, and polymeric pigments [40], where each represents a fraction in color of 50%, 33–42%, and 27–58%, respectively.

Contact with oxygen in red wines affects mainly anthocyanins and tannins. The degradation of anthocyanins shows an irreparable loss of color, with polymeric pigments being more resistant to oxidation, as well as those that do not have hydroxyl groups in the ortho position [22]. On the other hand, the condensation of anthocyanins with ethanal gives rise to red anthocyanin-ethyl-anthocyanin type compounds, which are not stable and may undergo further reactions with malvidin 3-glucoside or carboxypyrene-malvidin-3-glucoside, forming orange or blue pigments, respectively. The union of anthocyanins with other wine constituents leads to the formation of pyranoanthocyanins; moderate oxygenation benefits this formation [41]. The antioxidant potential of pyranoanthocyanins is maintained at the same level or even slightly increased compared to their respective anthocyanins; they also resist pH changes, hydration, and combine with SO_2_ better than their respective anthocyanins [29]. The most important pyranoanthocyanins, due to the role played by oxygen, are the vitisins B, presenting a hypsochromic shift with respect to the free anthocyanins. Hydroxyphenyl pyranoanthocyanins, such as pinotins, show an orange-red color (Figure 1); flavanol-pyranoanthocyanins have hypsochromically shifted from 490 to 511 nm; portisins present bluish-violet colors; oxovitisins have a yellow color [15,16]; methylpyranoanthocyanins cause a hypsochromic shift of the original anthocyanins, generating more orange [16]; and finally, the pyranoanthocyanin dimers present an attractive turquoise blue color with maximum wavelength (λ_max_) at 680–730 nm [36] (Figure 1). The anthocyanin-flavanol condensation in the presence of oxygen transforms the colorless compounds (A-F) to flavilium cations (A^+^-F) with intense red color, increasing the color intensity (CI) of the wine, which will be in equilibrium with the violet form (AO-F) and which will finally become yellow (400–500 nm) xanthanium salts [17]. These salts also form by the union of glyoxylic acid (oxidation of tartaric acid) or dehydroascorbate (oxidation of ascorbic acid), with flavanols giving colorless compounds that, by dehydration and oxidation, form orange-yellow xanthanium salts, with an increase in absorption at 440–460 nm. Oxygen also participates in the condensation of anthocyanin-flavanols with the participation of ethanal, forming violet pigments and adduct-like compounds linked by ethyl bridges, anthocyanin-ethyl tannins, which show a bathochromic shift of the anthocyanin with λ_max_ at 545 nm [34]. Oxygen also participates in the cross-polymerization of flavanols, forming high-molecular-weight polymers of yellow color, which are less reactive and decrease astringency.

Therefore, during controlled oxygenation of red wines, loss of absorbance at wavelengths of 505–530 nm is observed [7,42] by the decrease of monomeric anthocyanins, resulting in an increase of polymerized compounds (red pigments of the tannin-ethyl-anthocyanidin type or the anthocyanin-ethyl-anthocyanin type with a purple color) or yellow pigments of the xanthanium salts and new pigments such as pyranoanthocyanins (pigments mostly with a hypsochromic shift of the anthocyanin wavelength). The compounds formed show color stability, since they are more resistant to pH changes, to the influence of sulfur dioxide, and to decomposition (Figure 1). The addition of oxygen must be carried out in a controlled manner to avoid excessive formation of acetaldehyde, excessive polymerization, and precipitation of coloring matter. In addition to the amount of oxygen, the proportion of tannins and anthocyanins must be adequate, since too few tannins can lead to degradation of the anthocyanins, and too high a proportion leads to high polymerization and subsequent precipitation.

Hidden contact with oxygen during vinification is unavoidable [6]; thus, depending on the nature of the wine and the amount of oxygen incorporated, changes will occur that will determine the characteristics of the wine, and color is one of the most important. Therefore, it is necessary to have equipment that allows simultaneous measurement of the color variation of the wine and the consumed oxygen that has caused this evolution. This equipment will make it possible to know how much oxygen a wine can take on without altering its color, offering essential information for the winemaker regarding the care that must be taken with the wine to ensure its quality, thus avoiding undesirable surprises.

Therefore, the objective of this work was the validation and implementation of a device designed to perform a simultaneous and periodic measurement of the dissolved oxygen consumption kinetics in wine and its visible spectrum without interference during the process. The device was tested with commercial white and red wines to study the changes in their visible spectrum during oxygen consumption, allowing for the first time evaluation of the effect of oxygen at different moments of the consumption kinetics. The results show very interesting information, as it was found that wines in the early stages of oxygen consumption suffer a loss of absorbance in the visible spectrum and subsequently show absorbance increases that vary depending on the nature of the phenolic compounds in the wine. All red wines show a hypsochromic shift of their maximum wavelength after consuming specific amounts of oxygen, a shift that varies depending on the composition of the wine and whether it has been in contact with wood. This is very novel information, since no studies have described these changes during each moment of such consumption. The following work will focus on evaluating the effect of specific amounts of oxygen in certain areas of the ultraviolet visible spectrum, both in white and red wines, to see the influence of grape variety, origin, winery treatments, bottling, and corking to understand the response of the wines to specific amounts of oxygen.

## 2. Results

### 2.1. Characterization of the SpectrO_2_ unit

#### 2.1.1. Tightness Tests

**Figure 2:**
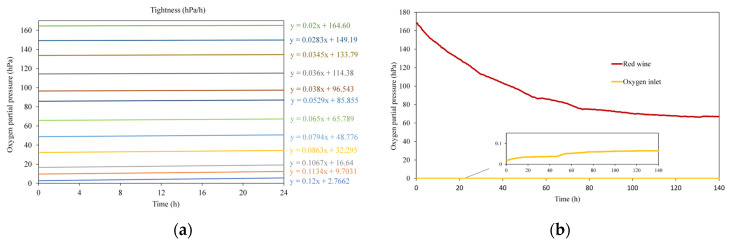
Tightness measurement. (**a**) Oxygen inlet in the 9 quartz cells filled with different levels of dissolved oxygen; (**b**) comparison of oxygen consumption of a wine and oxygen inlet in the quartz cell.

Figure 2a shows the average kinetics of oxygen entry into the nine quartz cells for each of the twelve oxygen levels studied, as well as the associated equation. Measurements were taken every 15 min for 24 h in each of the nine cells. The results show that the oxygen influx by diffusion, since the quartz cells were not pressurized, was dependent on the oxygen gradient inside the quartz cell with respect to the outside, such that the smaller the difference in oxygen concentration inside and outside the quartz cell, the lower the rate (slope) of oxygen influx. Previously published results [43] indicate that during the development of oxygen consumption kinetics, DO levels can vary from 165 hPa to 32 hPa, which would imply an oxygen inlet rate from air of 0.022 hPa/h to 0.086 hPa/h, respectively. The results show that for these quartz cells, when the gradient is close to maximum (DO = 3 hPa), an air O_2_ infiltration rate of 0.120 hPa/h occurs.

Figure 2b shows the average consumption kinetics of a red wine in the nine quartz cells and the oxygen inlet through the quartz cells depending on the concentration at each moment (oxygen inlet). It is observed that oxygen inlet accounted for between 0.02% (in the first 6 h) and 0.06% (in the last 72 h) of the oxygen contained in the quartz cells at the different time points of the consumption kinetics. These results indicate that the quartz cells were watertight, being suitable for monitoring the oxygen consumption kinetics of the wine they contain, even for the performance of tests where the partial pressure of oxygen was very low.

#### 2.1.2. Measurement of UV-Vis Spectra

**Figure 3:**
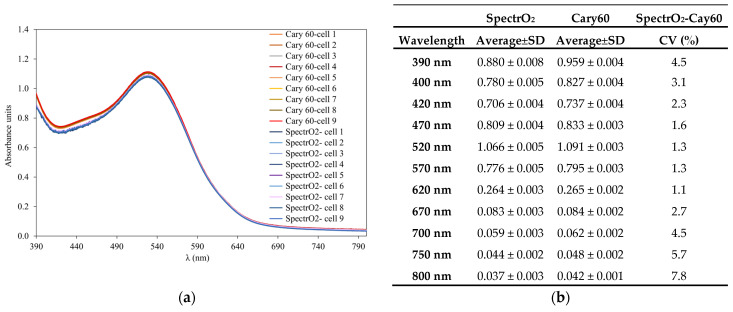
(**a**) Average spectrum of the 10 measurements within each quartz cell from 390 nm to 800 nm of the same red wine and (**b**) average absorbance (*n* = 10) at different selected UV-Vis lengths with SpectrO_2_ and Cary 60 UV-Vis equipment.

Figure 3 presents the average absorbance in the range of 390 nm to 800 nm of the same undiluted red wine measured in each of the nine 2 mm quartz cells ten times (*n* = 10) using the SpectrO_2_ equipment and commercial Cary60 equipment. The average absorbance at several selected wavelengths is also included, as well as the standard deviation of all the measurements taken in all the quartz cells (10 measurements in each of the nine quartz cells, *n* = 90). The SpectrO_2_ results show that this equipment allows quantifiable measurements over the entire range of the spectrum studied. The absorbance results of a red wine at the selected wavelengths varied between 0.037 and 1.066 units, with a standard deviation ranging between 0.002 and 0.008, showing in all cases a coefficient of variation of less than 7%.

The spectra obtained with the two types of equipment (SpectrO_2_ and commercial Cary60) were very similar (Figure 3b), showing absorbance differences in the spectra of less than 0.042. The coefficients of variation of the absorbance obtained with both devices in the range of 400 nm and 670 nm were from 1.1 to 3.1%; between 700 nm and 800 nm the coefficients of variation varied between 4.5% and 7.8%. The results demonstrate the potential of the SpectrO_2_ device to perform spectral scans from 390 nm to 800 nm (a range of great interest in oenology), without interference from the optoluminescent DO measurement system. Therefore, the SpectrO_2_ device made it possible to follow the kinetics of oxygen consumption and, in addition, to simultaneously measure the color without manipulating the sample so as not to expose it to the air while the wine was consuming oxygen. All this demonstrates the potential, precision, and accuracy of measuring the visible spectrum simultaneously with the optoluminescent measurement of DO with the SpectrO_2_ device.

#### 2.1.3. Measurement of Oxygen Kinetics

The first row of Table 1 collects the 13 parameters calculated according to del Alamo-Sanza et al., 2021 [43], to define the oxygen consumption kinetics of the same red wine analyzed in the nine quartz cells simultaneously (*n* = 9) using the SpectrO_2_ equipment. The parameters giving information on oxygen content (*O_max_*, *O_min_*, ∆*O_max_min_*, *O_int_*, *O*_90_, ∆*O*_90_10_ and *O*_10_) showed that with the use of the different quartz cells, results with a coefficient of variation of less than 3% are obtained. At the beginning of the kinetic process, the differences in *O_max_* in the wine of the nine quartz cells was less than 2.1 hPa (coefficient of variation of 1.1%), while when evaluating the end of the oxygen consumption (*O_min_*), after 147 h, the difference between the nine quartz cells was 1.8 hPa (coefficient of variation of 2.9%). Regarding the time-related parameters (*t_O_min_*, *t*_*O*_90_, *t*_*O*_10_, and *t_O_int_*), differences were found that reflect variations in the time required to consume certain amounts of oxygen in each cell, differences of less than 1 h to consume 10% of the available oxygen (*t*_*O*_90_) and 3.1 h to reach the residual oxygen (*t_O_min_*). The greatest differences were found in the time parameter, with the time taken to consume the first 10% of oxygen (*t*_*O*_90_) being the parameter that presented the greatest coefficient of variation (15.9), with a difference of 0.7 h between the nine quartz cells. Finally, the parameters related to the area under the curve (*AO_max_min_* and *A_max_min_*) showed differences of less than 3.5%. The described differences indicate that the kinetics of oxygen consumption in all the quartz cells are the same, so that the performance of this monitoring is reproducible in all the quartz cells of the equipment.

### 2.2. Measurements of Commercial Wines with SpectroO_2_

Different wines were evaluated with the SpectrO_2_ device to determine their ability to discriminate between different regions of the spectrum and different times of DO consumption kinetics. A young wine (red wine 1) of the Tinta de Toro variety has 14.5% vol, a high concentration of free anthocyanins (447 mg/L), and a balanced level of low and highly polymerized phenols (397 and 206 mg/L, respectively), which define a CI of 13.62, showing a high contribution of the red component (51%), expressed as %520 nm. A young wine of the Mandon variety (red wine 2) has a low0 alcohol content of 10.51% vol., a free anthocyanin content of 197 mg/L, and a low concentration of total phenols (468 mg/L), of which 83% are polymerized. This wine had a CI of 7.69, with a significant red component (52%), typical of young wines as in red wine 1. On the other hand, a barrel-aged wine (red wine 3), made from Cabernet Sauvignon, had an alcohol content of 13.5% vol., a CI of 11.25, and a weight of 41% for the yellow tones (%420 nm), similar to 46% of the red tones (%520 nm). This wine had a lower level of free anthocyanins (74 mg/L) and a higher level of total phenols (670 mg/L), evenly divided between slightly and highly polymerized phenols, at 56% and 44% respectively. With respect to the white wines, two wines with very similar total acidity, pH, alcohol content, and phenolic load were studied: white wine 1 was a young wine made from Sauvignon blanc, and white wine 2 was a barrel-fermented Verdejo variety, having almost twice the CI of white wine 1. Both white wines showed a high level of T-SO_2_ versus low F-SO_2_ content, with the latter being especially low in white wine 2; the red wines did not show free or total SO_2_.

**Figure 4:**
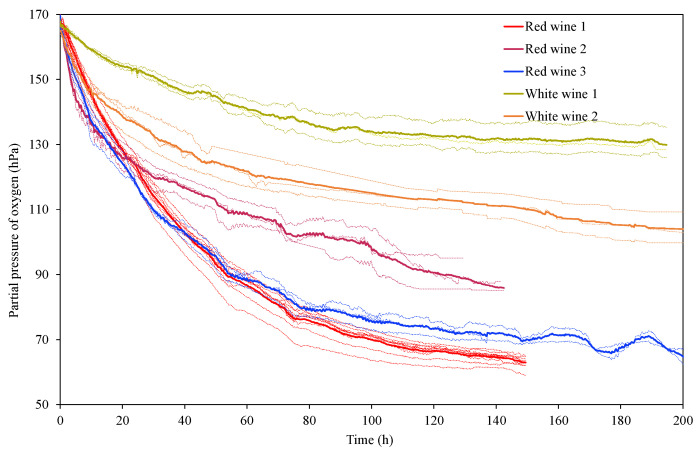
Oxygen consumption kinetics of the red wines and white wines described in Table 1. The dotted line indicates each replicate and in solid line the mean.

Figure 4 shows the oxygen consumption kinetics of the five wines, and the parameters describing their kinetics are shown in Table 1. The dissolved oxygen reached by the wines when subjected to saturation (*O_max_*) reached oxygen partial pressure pO_2_ values close to 168 hPa and did not present significant differences between white and red wines, results that coincide with what has been previously described [8,43]. The same occurs with the time the wines needed to consume the first 10% of oxygen (*t*_*O*_90_), with average values of approximately 3 h, or with the time it took to drop to 50% of the oxygen level reached after saturation with air (*t_O_int_*), which was 28 h, showing no differences between white and red wines. However, the time to reach the end of the consumption kinetics, i.e., the time needed for the wines to consume all the oxygen they were capable of consuming (*t_O_min_*), differed slightly between whites and reds, with white wines needing on average 199 h (8 days), and red wines 162 h (7 days). Regarding the oxygen level that red and white wines showed in the different sections of the consumption kinetics, it started to differ after the consumption of the first 10% of oxygen (*O*_90_), i.e., *O_int_*, *O*_10_, *O_min_*, ∆*O*_90_10_, and ∆*O_max_min_*. Thus, it is observed that red wines were able to consume more oxygen than white wines, with an average total consumption of 98 hPa (∆*O_max_min_*), while in white wines it was 50 hPa. Therefore, the residual or minimum oxygen (*O_min_*), i.e., the oxygen that they were not able to consume, was much higher in white wines than in red wines, with values of 116 hPa and 67 hPa, respectively, a difference of 50 hPa. Thus, the red wines studied consumed more oxygen in less time, which demonstrates greater oxygen avidity than the white wines and is reflected in the lower areas under the consumption kinetics curve (*AO_max_min_* y *A_max_min_*). The analysis of the red wines indicates that red wine 2 presented a lower oxygen consumption (*AO_max_min_*), at 81 hPa, compared to 106 hPa and 108 hPa for red wine 3 and red wine 1, respectively. Thus, red wine 2, wine with lower phenolic, alcohol, and CI content, presented a residual oxygen (*O_min_*) of 85.9 hPa, higher than the 81.3 hPa it was able to consume (∆*O_max_min_*). All white wines presented the same situation, an *O_min_* greater than ∆*O_max_min_*; the white wine with wood, the wine with the higher CI and alcohol and lower SO_2_ and the one that was able to consume the highest oxygen content, consumed 24 hPa more than white wine 1.

**Figure 5:**
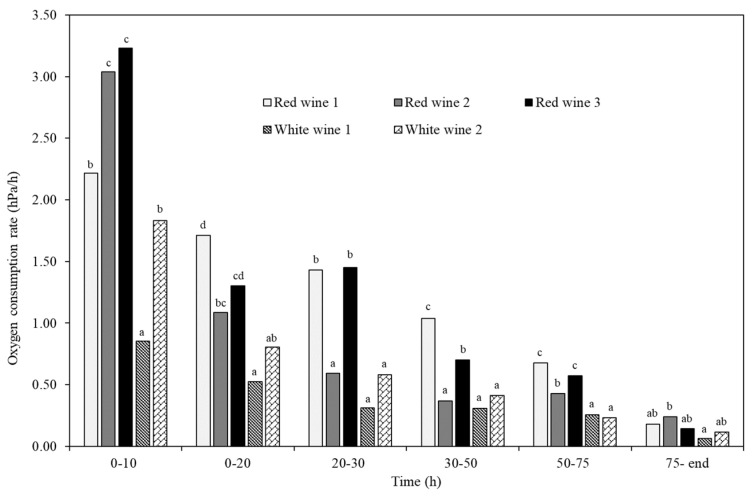
Modification of the oxygen consumption rate of different wines over time. Different letters indicate significant differences between wines at same time.

Therefore, the composition of the wines defines their oxygen avidity (Figure 5, Table 1), with each of the wines presenting a different oxygen consumption rate, especially in the first phases. This result made it possible to define seven sections with different oxygen consumption rates in all the wines: a first phase from the beginning until 10 h; a second until 20 h; a third phase until 30 h; a fourth from 30 h to 50 h; a fifth from 50 h to 75 h; and the last phase, which ended when the wine reached the minimum or residual oxygen (*O_min_*), which varies according to the type of wine, from 136 h to 205 h. These sections confirm what has been described in other works, which is that, in general, red wines initially present a higher consumption speed than white wines due to their higher content of phenolic compounds [3,9], although depending on the type of wine, very similar behaviors can be found between white and red wines [8,40,43].

Figure 5 presents the oxygen consumption rate of each wine in the different periods studied. The highest rate of oxygen consumption in all the wines occurred in the first period, a rate that decreased during the kinetics, being lower with lower amounts of available oxygen. Red wine 1, young Tinta de Toro, started consuming oxygen at 2.2 hPa/h and ending at 0.2 hPa/h, with a rate of 3.6 mg/L over 150 h; red wine 2, young Mandón, consumed 2.8 mg/L of oxygen in six days at a rate that started at 3.2 hPa/h and ended at 0. 2 hPa/h; and red wine 3, barrel-aged Cabernet Sauvignon, showed an avidity of consumption like red wine 1, of 3.7 mg/L, which started at 3.23 hPa/h, and thus was faster than red wine 1 but was slower than red wine 1 after 10 h, ending at a consumption of 0.1 hPa/h, which resulted in the longest consumption kinetics. In the first 10 h, the young red wines consumed 2.21 and 3.04 hPa/h, and in the first 20 h they consumed 38% and 51% of all the oxygen they were able to consume (3.6 and 2.8 mg/L). However, the barrel-aged red wine, red wine 3, started consuming at a higher rate (3.23 hPa/h, first 10 h) than red wine 1, consuming in 20 h 43% of all the oxygen it was able to consume (3.7 mg/L), consumption that quickly slowed down, ending the kinetics two-and-a-half days later than red wine 1 (Figure 5).

This result may be because the compounds released by the wood interact with the wine’s own compounds, forming new compounds with greater oxygen avidity. In the case of white wines, in the first 10 h, the oxygen consumption of the aged white wine was also much faster than that of the young wine, at 1.83 hPa/h vs. 0.85 hPa/h. Subsequently, the rate of consumption was more similar between the two wines, and although a reduction in rate was observed in the aged white wine 2, the wine consumed more oxygen. It was found that white wines consumed less oxygen and much more slowly than red wines, so the percentage of total oxygen that white wines and red wines were able to consume in the first 20 h was similar; for the white wines, this percentage was 36% and 43% in white wine 1 and white wine 2, respectively. It was also found that red wines consumed between 82–88% of all oxygen in the first 75 h, while white wines consumed between 76 and 78%, indicating a longer period of low oxygen consumption rate. It should be noted that both white wines and red wines in contact with wood (red wine 3 and white wine 2) showed longer consumption kinetics than red wine 1, red wine 2, or white wine 1.

**Figure 6:**
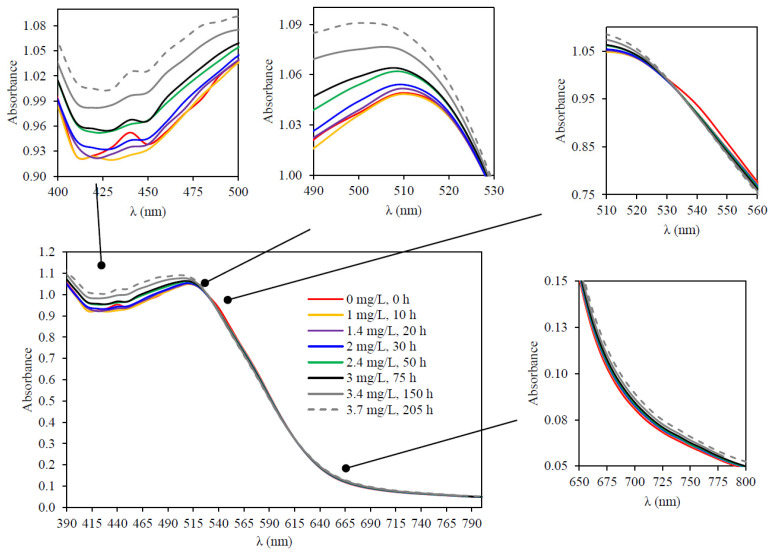
Evolution of the red wine 3 spectrum during oxygen consumption at 8 different times.

**Figure 7:**
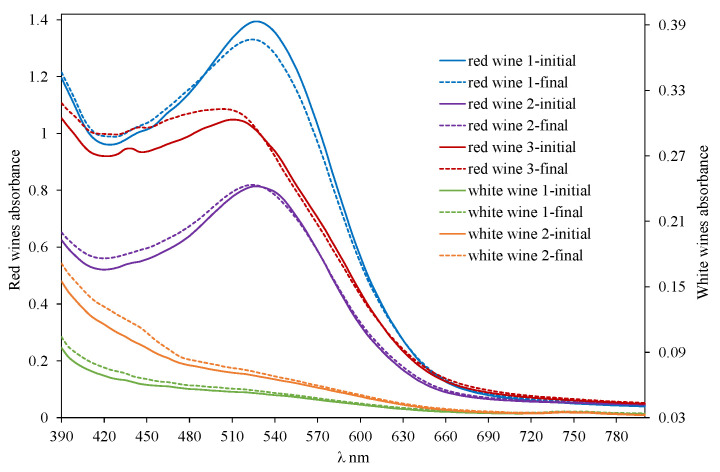
Spectrum of the red (left axis) and white wine (right axis) before (initial) and after (final) oxygen consumption.

The most interesting capability of the SpectrO_2_ device is the possibility of simultaneously measuring the kinetics of oxygen consumption and the wine spectrum, which makes it possible to evaluate the changes that occur in the spectrum due to the consumption of specific amounts of oxygen at specific times of the consumption kinetics. With respect to the evolution of the wine spectrum as oxygen is consumed, Figure 6 presents the modification of the red wine 3 spectrum at eight different times corresponding to the seven sections described above and to the initial spectrum. The modification of the spectra of the other wines studied is presented in Supplementary Figure S1 (a—red wine 1; b—red wine 2; c—white wine 1; d—white wine 2). In each figure, the oxygen consumed by each wine for each of these moments is indicated, enlarging the area of the spectrum in which some significant event occurred by increase or decrease of absorbance or change of trend. It is expressed in mg/L, considering the atmospheric pressure of each measurement and assuming that the solubility of oxygen in wine is the same as in water. Figure 7 shows the variation of the spectra of the five wines studied before consuming oxygen and after having consumed all the oxygen they are capable of consuming. The variation in the behavior of the different wines allows us to anticipate that oxygen consumption implies very different spectral variations for each type of wine, a spectral fingerprint that contains information that is what the developed device is intended to determine.

**Figure 8:**
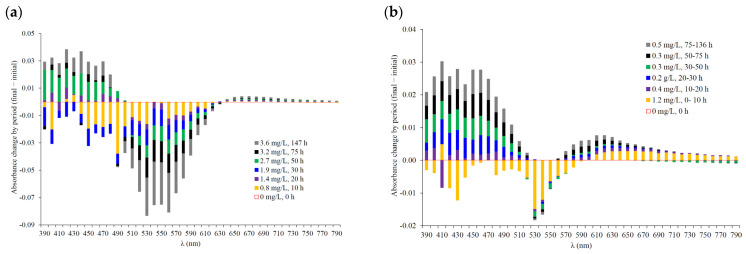

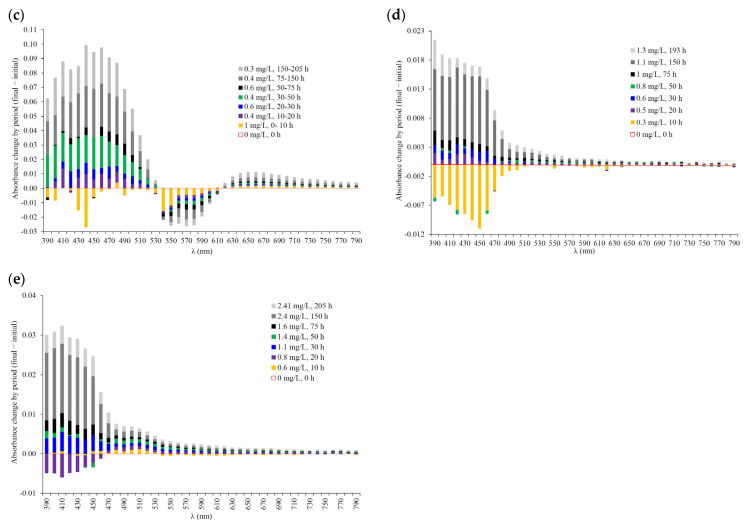
Change in absorbance during oxygen consumption at each wavelength, corresponding to each period studied (absorbance at end of period minus that at beginning of period), as well as the oxygen consumed in that period: (**a**) red wine 1; (**b**) red wine 2; (**c**) red wine 3; (**d**) white wine 1; (**e**) white wine 2.

The study of the spectral variation of red wines during the seven selected moments of oxygen consumption kinetics indicated that, initially, the maximum absorbance was between 525 and 535 nm (red wine 1 and 2) and 505 and 515 nm (red wine 3) (Figure 8). The device makes it possible to analyze the variation of the spectrum of a wine and to analyze the different behaviors of oxygen consumption between wines; for example, the initial maximum of red wine 3 had a hypsochromic shift with respect to red wine 1 and red wine 2. This result may be due to the evolution of its color during its aging in barrels and subsequent time in bottles; thus, in this wine, an important contribution of the absorbances between 400 and 500 nm was observed (Figure 7) as well as a greater contribution to its color of the absorbance at 420 nm. This defines the intense red color of the young wines (red wine 1 and 2) and the brick red of the aged wine (red wine 3). It is observed that oxygen consumption led to a hypsochromic shift of the initial maximum of the visible spectrum in all red wines. At the end of the oxygen consumption kinetics, the λ_max_ in red wine 1 and red wine 2 was at 515–520 nm (10 nm less) (Supplementary Figure S1a,b), with the displacement of red wine 2 (Figure 6) wine being more pronounced with lower initial absorbance; in red wine 3, the displacement was 20 nm, showing a λ_max_ at 485–490 nm (Figure 6). If this analysis of spectral variation is applied to the white wines, within the range of the spectrum studied, they presented the maximum absorbance at 390 nm, both before and after oxygen consumption. In both white wines, the absorbance at 390 nm increased with oxygen consumption and in the same proportion, being 61% higher in white wine 2 than in white wine 1 before and after oxygen consumption (Figure 7).

Table 2 presents the change in absorbance with respect to the beginning of each period studied for each wine, Table 3 shows the correlation for each wine between oxygen consumed and absorbance at different wavelengths, and Figure 8 shows for each wine the change in absorbance for each wavelength, differentiating each time period studied (absorbance at the end of the period minus that at the beginning of the period). Oxygen consumption caused changes in the color of the wines, and it is interesting to analyze at different times how oxygen affects the different zones of the spectrum.

In general, at the beginning of the oxygen consumption kinetics, the absorbance of red wines decreased; however, there were changes in trends at different wavelengths, depending on the type of wine. Thus, it is observed that the effect of oxygen caused an increase and loss of absorbance in different sections of the range studied (390 nm to 800 nm). From 390 to 480 nm, the absorbance of red wines increased with oxygen consumption in red wine 1, up to 533 nm in red wine 2, and up to 532 nm in red wine 3. However, from 480 nm in red wine 1 and from 533 nm and 532 nm in red wine 2 and red wine 3, respectively, with oxygen consumption there was a trend change, and the absorbance of the wines decreased up to 630 nm, 564 nm, and 616 nm in red wine 1, 2, and 3 respectively; from these wavelengths, another trend change occurred, increasing the absorbance up to 750 nm (Figure 6 and Figure 8 and Supplementary Figure S1a,b).

These results indicate that the consumption of 1.9 mg/L oxygen by the wine with the highest CI (red wine 1) caused an increase in absorbance from 390 nm to 490 nm, and at the end of the consumption kinetics, the most significant increase, up to 0.035 units (Table 2, Figure 8a), occurred between 420 nm and 440 nm. It also showed a lower absorbance increase range (390 nm to 480 nm) than that found for red wine 2 or red wine 3, which showed absorbance increase ranges from 390 nm to 533 nm and 532 nm, respectively. The greater increase in the orange tones of red wines 2 and 3 compared to red wine 1 may be due to the fact that since they have a lower anthocyanin content, oxygen is more present in reactions in which they are not involved, such as the polymerization of flavanols, the binding of flavanols to glyoxylic acid or ascorbic acid, etc., causing these wines to evolve more in these wavelengths. Therefore, with oxygen consumption, red wines 2 and 3 underwent color evolution more towards oxidation-related tones (Figure 7), especially red wine 3, which contains more flavanols due to its barrel aging. These results are reflected in the correlations between the oxygen consumed by the wines and the absorbance in this area of the spectrum, which are positive in all the wines, with red wine 3 showing the highest weights and significance followed by red wine 2 (Table 3). This increase in absorbance may be related to the completion of the formation reactions of colorless compounds, such as the condensation of anthocyanins-flavanols, flavanols-flavanols, and glyoxylic acid-flavanols, in addition to the possible degradation of certain compounds, such as anthocyanins [29]. These colorless products are intermediates of many of the reactions that occur when wine is exposed to oxygen, forming c compounds that absorb in this wavelength range, such as xanthanium salts, the binding of anthocyanins linked to oligomeric methylmethine, in addition to the formation of new compounds such as pyranoanthocyanins, which mostly form complexes of orange hues [25,44,45].

However, oxygen consumption caused a loss of absorbance in red wine 1, between 480 nm and 630 nm, which was greater than that suffered by red wine 2, with a decrease between 533 nm and 564 nm, or red wine 3, with a decrease between 532 nm and 616 nm. In the case of red wine 1, the absorbance between 480 nm and 630 nm decreased with oxygen consumption from the beginning until the end of the kinetics, presenting a loss of up to 0.1 absorbance units at 530 and 560 nm (Figure 8a) (6 and 7%, respectively, with respect to the beginning; see Table 2). The most relevant loss occurred in the interval from 520 nm to 580 nm at the end of the kinetics, when red wine 1 consumed 0.4 mg/L (Figure 8a). This ability to compare variations in the spectrum with oxygen consumption is one of the strengths of the device used and allows the behavior of each wine to be analyzed. This result may suggest that the higher content of free anthocyanins in red wine 1 facilitated their interaction with oxygen, causing both the oxidation and loss of these anthocyanins, as well as the formation of new pigments, reflected in the loss of absorbance in this range. In red wine 2, absorbance losses were only observed throughout the kinetics in the 540–560 nm range; specifically, from 500 to 530 nm, the wine lost absorbance with the consumption of the first mg/L of oxygen, but later, when another 2.1 mg/L of oxygen had been consumed, it was observed that after 50 h the absorbance increased with respect to the initial absorbance (Table 2). Similar results were found from 570 nm to 590 nm, although in this section it was not until the consumption of the last 0.5 mg/L (137 h) that the increase in absorbance with respect to the initial spectrum was observed (Table 2). Therefore, in red wine 2, significant negative correlations were observed only with oxygen consumption and absorbance loss at 540–555 nm (most significant between 545 nm and 550 nm; −0.8718 and −0.8907, respectively). This result may indicate that the lower anthocyanin content of red wine 2 leads to fewer reactions with oxygen than in red wine 1, hence the lower absorbance loss (−0.5 to −1.6%) at these wavelengths (Table 2 and Table 3). On the other hand, red wine 3, a barrel-aged wine with the lowest content of free anthocyanins, showed the lowest absorbance losses (0.1%) in the range of 500 to 520 nm, with the consumption of 1 mg/L (Table 2), possibly due to the stability of its coloring matter by the formation of compounds in the wine with those in the wood. Red wine 3, after consuming 2 mg/L, presented higher absorbance than the initial wine, showing the greatest increase with the consumption of the last 0.7 mg/L of oxygen, which accounted for the greatest increase in absorbance at 500 nm (5.2%). These results were reflected in the positive correlations between the absorbance of red wine 3 at the different times studied and the oxygen consumed, with the most significant correlation at 515 nm (0.9054) (Table 3), which may explain the hypsochromic shift mentioned above. The effect of oxygen consumption of red wine 1 versus red wines 2 and 3 in this area of the spectrum could be due to the higher level of polymerization of the phenolic compounds of red wines 2 and 3. The oxygen consumption in red wine 3 caused a 3–4% loss of absorbance from the beginning to the end of consumption from 540 to 610 nm (not reaching 0.03 units, Figure 8c), lower than in red wine 1, which lost 7% (0.085 units) absorbance in the range of 540–560 nm (Table 2). In red wine 3, it is also observed that the loss of absorbance occurred in different zones according to the oxygen consumed, showing that in the first 75 h, when the wine consumed 3 mg/L, the greatest loss was between 540 nm and 580 nm, while the consumption of the last 0.7 mg/L of oxygen caused a greater loss of compounds, with absorbance between 570 nm and 590 nm. Therefore, the correlations between oxygen consumed and absorbance in this range of the spectrum were negative (Table 3), having more significance from 550 nm to 605 nm; however, despite their high significance, they were lower than those found in the young wine with high anthocyanin content (red wine 1).

Regarding the purple-violet range of the spectrum and up to the 800 nm studied, oxygen consumption caused an increase in absorbance. In the case of red wine 1, the absorbance increased in the 640 to 700 nm range throughout the consumption kinetics, and although the consumption of the first 0.8 mg/L of oxygen caused a loss of absorbance from 710 to 800 nm, it subsequently increased with respect to the initial absorbance, needing to consume 1.4 mg/L and 2.7 mg/L of oxygen to reach an absorbance higher than the initial absorbance between 710 to 730 nm and between 740 to 780 nm, respectively (Table 2, Figure 8a). Thus, oxygen consumption showed significant positive correlations with absorbance up to 750 nm, with the highest weights from 640 to 700 nm (Table 3). In red wine 2 and red wine 3, an increase in absorbance at these wavelengths was also observed, higher than in red wine 1, from 610 and 620 nm to 800 nm in red wine 2 and red wine 3, respectively (Table 2 and Figure 8). The increase in absorbance at these wavelengths due to oxygen exposure can be explained by the formation of new compounds that absorb at these wavelengths, such as portisins, pyranoanthocyanins dimers, and carboxypyrane-malvidin-3-glucoside [29]. In red wine 2, it was observed that the greatest increase in absorbance occurred from 680 nm to 710 nm with the consumption of the first 1.2 mg/L of oxygen and also from 640 to 690 nm with the consumption of the last 0.5 mg/L; this increase at 660 nm was 13.9% with respect to the initial wine (Table 3, Figure 8b), with a positive correlation throughout the range, although only significant at 690 nm (+0.7815) (Table 2). However, the greatest increase in absorbance in red wine 3 occurred when the rate of oxygen consumption decreased considerably from 0.57 to 0.14 hPa/h, i.e., with the consumption of the last 0.7 mg/L of oxygen (Table 2). Red wine 3 underwent the greatest increase in absorbance, from 670 to 750 nm, an increase of 10.7% with respect to the initial wine (Table 2), showing a significant and positive correlation with oxygen consumption, standing out at 690 nm (+0.927).

Therefore, the effect of oxygen on the absorbance of the wines in this zone of the spectrum was quite different between red wine 1 and red wines 2 and 3, being more significant in the latter two and especially in red wine 3. This may be due to the type and level of polymerization of the phenolic compounds of red wine 3; possibly, this wine presents compounds derived from the reactions of the wine compounds with those yielded by the wood, compounds different from those of a young wine, which, as described throughout the work, cause the consumption of oxygen by red wine 3 to produce a different behavior in the evolution of its color. In the case of young red wines, the differences in the effect of oxygen consumption on color evolution are related to the nature of the phenolic compounds, since red wine 2 had a lower phenolic content, with 83% of its phenols highly polymerized compared to 34% in red wine 1 (Table 4).

In the case of the white wines, the effect of oxygen consumption on the spectrum shows two completely different profiles (Figure 8d,e), although globally, in both wines, it causes an increase in absorbance, which is more evident at 390–450 nm (Table 2) and has more significant correlations between the increase in absorbance and oxygen consumption at 505–515 nm (Table 3). These wines consumed less oxygen than the red wines (1.30 mg/L and 2.41 mg/L) and more slowly (Figure 5). White wine 1, a young wine that consumed in the first 10 h 0.3 mg/L at a rate of 0.8 hPa/h, suffered a loss of absorbance from 410–450 nm, specifically 10% and 16% with respect to the initial absorbance at 410 and 440 nm, respectively (Table 3) (0.009 and 0.11 absorbance units). After 10 h, oxygen consumption caused an increase in absorbance, being more important between 390 and 460 nm (Figure 8d); at the end of consumption, it was up to 19.4%, at 450 nm (0.015 absorbance units), higher than that of the initial wine (Table 2). The most important increase was observed when consumption slowed down from 0.26 to 0.06 hPa/h, i.e., when it consumed the last 0.3 mg/L (Figure 8d), showing an absorbance higher than the absorbance of the initial wine (Table 2). Therefore, the spectral change in the 390–460 nm range of white wine 1 occurred with the consumption of the first and last 0.3 mg/L of oxygen, decreasing and increasing, respectively (Figure 8d); thus, there were no correlations between oxygen and absorbance change (Table 3). The decrease in absorbance was probably due to the formation of colorless components such as xanthenes, whereas the increase in absorbance could have been due to the completion of chemical reactions initiated by the formation of, for example, xanthanium salts. The oxygen consumption of white wine 2 shows a very different profile, probably due to the presence of wood compounds extracted during the barrel fermentation process. Both wines showed an initial decrease between 390 and 460 nm, being lower and later in white wine 2, which needed to consume 0.8 mg/L (Figure 8e) to reduce absorbance by 2–4% (Table 2). When white wine 2 had consumed more than 0.8 mg/L, it showed an increase in absorbance at 390–460 nm, reaching more than 20% at the end of consumption (Table 2) and showing a much higher browning than white wine 1, with an increase of 0.03 absorbance units versus 0.018 in white wine 1 (Figure 7 and Figure 8d,e). The increase of yellow tones in white wines due to contact with oxygen is a well-known fact due to the oxidation of phenolic molecules and their corresponding quinones; to the polymerization of flavonoids, forming brown pigments; and to the oxidation of tartaric acid and ascorbic acid, which reacts with flavanols, forming colorless compounds that by oxidation turn to yellowish-brown pigments [10,11,12,13]. The difference between white wine 1 and white wine 2 may be because the main phenolic compounds in the former are hydroxycinnamic acids, and in the latter flavanols, compounds related to young wines and wines in contact with wood, respectively. The involvement of flavonols with oxidation has been previously reported, while hydroxycinnamic acids are implicated in the presence of flavanols [12]. Therefore, white wine 2 shows higher susceptibility to browning, presenting a golden color and a positive and significant correlation with oxygen (Table 3).

In white wine 1, the greatest increase in absorbance was observed with the consumption of 0.5 mg/L in the first 20 h at 510 nm, which moved to 450 nm when the wine consumed more than 0.6 mg/L. In white wine 2, the greatest increase in absorbance occurred between 480 nm and 510 nm in the first 75 h, with the consumption of 1.6 mg/L; when the wine consumed the last 0.81 mg/L, the increase in absorbance moved to 430–450 nm (the stretch from 75 to 205 h). White wine 2 consumed 2.41 mg/L oxygen in 205 h at an initial oxygen consumption rate of 1.83 hPa/h, reflecting more avidity than white wine 1, at 0.85 hPa/h, a rate that remained higher throughout the consumption kinetics. In white wine 1, the absorbance did not increase due to oxygen consumption; however, white wine 2, when it had consumed 1.4 mg/L of oxygen (50 h), presented in the whole range studied more absorbance than initially, showing a significant correlation, especially in the stretch from 480 nm to 530 nm. These results reflect the changes previously described in white wines subjected to oxidative changes, and although the compounds responsible are unknown, a possible explanation is that they come from the hydrolysis of leucoanthocyanins in the corresponding flavilium salts or from the reaction of the glutathione reaction compound with o-quinones, which produces browning pigments [12].

## 3. Materials and Methods

Equipment for the simultaneous measurement of oxygen consumption and wine color, called SpectrO_2_, was designed, built, and validated. To analyze the performance and capabilities of the new equipment, 5 different wines were studied.

### 3.1. Wines Analyzed

Table 4 lists the characteristics of each of the wines, including grape variety and vintage. Total acidity (g/L tartaric acid), pH, alcoholic strength (% vol), free sulfur dioxide (F-SO_2_, mg/L), total sulfur dioxide (T-SO_2_, mg/L) were measured following the methods established by International Organization of Vine and Wine [46]. Color parameters were determined by a direct measurement of wine absorbance at 420, 520, and 620 nm in a 1 mm or 10 mm optical path length quartz cell for red and white wines, respectively, with PerkinElmer’s LAMBDA 25 UV/vis Spectrophotometer (Waltham, MA, USA). CI and red, yellow, and blue percentages were also calculated following the method described by del Alamo Sanza et al., 2004 [47]. The total phenol index (TPI) was measured after adequate dilution in a 10 mm optical path length quartz cell using the same spectrophotometer. The total phenols (TPs), low polymerized phenols (LPPs) and high polymerized phenols (HPPs) as mg/L of gallic acid, total anthocyanins (ACY, as mg/L of malvidin-3-*O*-glucoside) were analyzed according to the methods described by del Alamo Sanza et al., 2004 [47].

### 3.2. SpectrO_2_ Device

**Figure 9:**
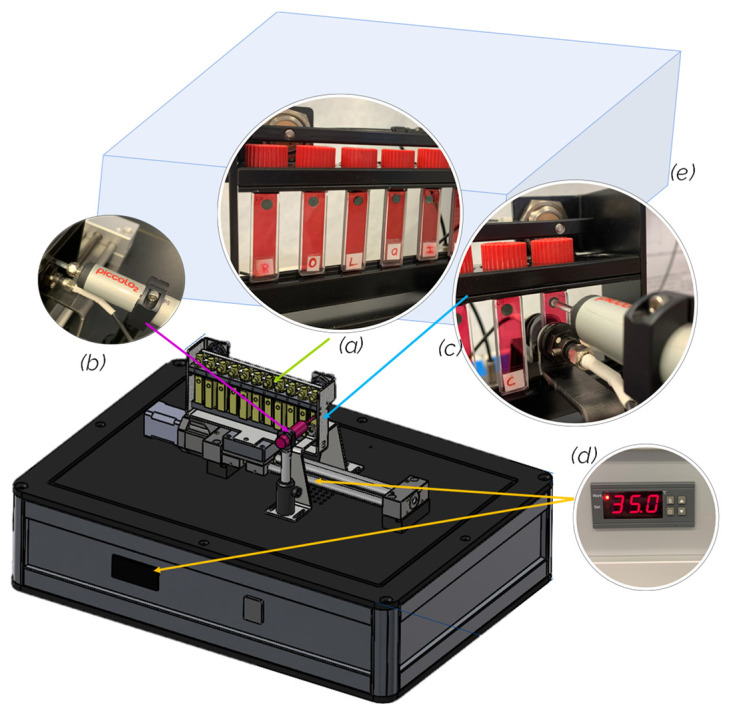
Device for simultaneous measurement of wine oxygen consumption and wine spectra: 9 quartz cells (**a**), dissolved oxygen meter (**b**), spectrophotometer (**c**), temperature control system (**d**), and cover (**e**).

The device has 9 quartz cells of 2 mm thickness. It is closed with a screw cap fitted with a butyl septum and placed in a frame on a 9-position rail with an automated system for moving the frame to perform the spectrophotometric measurements (Figure 9). The high-precision oxygen measurement system consists of a model Piccolo2-OEM detector (PyroScience GmbH, Aachen, Germany), which allows measurement of the partial pressure of dissolved oxygen (DO) in liquid and in gas and which is equipped with an external temperature sensor. The oxygen sensors glued inside each cell are PSt3 Oxygen-Sensitive Spots (PreSens Precision Sensing GmbH, Regensburg, Germany), which are calibrated before each use according to the manufacturer’s protocol at two levels, 0% air sat. and 100% air sat. The spectrometer is an Ocean Optics HR4000 (Ocean Optics, a Halma Company, Dunedin, FL, USA), with high optical resolution, equipped with a high-sensitivity 3648-element CCD array detector from Toshiba, which allows absorbance measurement in the range of 390 nm to 800 nm. The device is equipped with a Peltier-type temperature control system, which allows the working temperature to be regulated and maintained with an accuracy of 0.1 °C. The whole system is controlled by a software developed in Labview (National Instruments Corporation, Austin, TX, USA), which allows management of the device as well as programming of the measurements and storage.

### 3.3. Evaluation of Cell Quartz Tightness

The cells must be leak-tight to prevent the ingress of atmospheric oxygen and to avoid interference during the measurement of DO consumption kinetics. Tightness was evaluated by filling the quartz cells with a gas mixture (air and nitrogen, Carburos Metálicos, Air Products Group, Barcelona, Spain) with a known oxygen concentration. The gas mixtures were prepared with the Gm-3 gas mixer (Sensor Sense, Nijmegen, The Netherlands). The tightness of the quartz cells with wine was evaluated at different levels of dissolved oxygen, between the maximum content reached after saturation with air and the lowest dissolved oxygen content reached once the consumption kinetics, as detailed in a previous work [43], ended. The tightness of the 9 quartz cells was evaluated for 24 h at 12 different DO levels, from 165 hPa to 3 hPa.

### 3.4. Evaluation of Repeatability

#### 3.4.1. Spectrum Repeatability

The evaluation of the repeatability of the spectrum measurements in the range of 390 nm to 800 nm was performed by measuring the same wine 10 times for each of the 9 quartz cells of the SpectrO_2_ equipment. The results were compared by measuring the same wine within the same quartz cells using a commercial spectrophotometer, Cary60 UV-Vis (Agilent Technologies, Santa Clara, CA, USA).

#### 3.4.2. Repeatability of Consumption Kinetics

The evaluation of the repeatability of the consumption kinetics measurements was carried out by measuring the same wine in the 9 quartz cells simultaneously. The wine was treated according to the procedure of del Alamo-Sanza et al., 2021 [43]. The samples were briefly tempered at 35 °C and saturated with air at 35 °C by bubbling with a ceramic microdiffuser at a flow rate < 1 mL/min for a period of 5 min. Immediately, the saturated wine was transferred to the 9 quartz cells of the SpectrO_2_ device to evaluate the kinetics of oxygen consumption by measuring dissolved oxygen every 15 min until the end of oxygen consumption. The working temperature was 35 ± 0.1 °C, following the method of Nevares et al., 2017 [8].

### 3.5. Simultaneous Measurement of Oxygen Consumption Kinetics and Spectra with SpectrO_2_ Equipment

Samples were prepared for the measurement of consumption kinetics as described above. Each test allowed the measurement of 3 wines simultaneously in triplicate, filling the 9 quartz cells available. Dissolved oxygen measurements were performed every 15 min until the end of the oxygen consumption kinetics and, simultaneously, measurements of the visible spectrum between 390 and 800 nm were also taken every 15 min. Simultaneous monitoring was carried out until the end of the oxygen consumption kinetics, which, depending on the type of wine, varied between 6 and 9 days.

### 3.6. Oxygen Consumption Kinetics Processing

The oxygen consumption kinetics data were processed according to del Alamo-Sanza et al., 2021 [44]. Briefly, the data from each of the 3 repetitions of the consumption curve were processed to obtain the highest value of dissolved oxygen, identified as the beginning of the kinetics, and the lowest value, which indicates the end of the consumption kinetics. These curves were fitted to the inverse fitting model because it is the best method to describe and extract characteristic parameters of the oxygen consumption kinetic curves. The parameters correspond to *O_max_*: maximum/initial oxygen value (hPa); *O_min_*: minimum/residual oxygen value (hPa); ∆*O_max_min_*: total oxygen consumed (hPa); *O_int_*: oxygen value when half of the total oxygen has been consumed (hPa); *O*_90_: oxygen representing 90% of the range between the maximum and minimum values, i.e., when the first 10% of total oxygen (hPa) has been consumed; ∆*O*_90_10_: variation between 90% and 10% oxygen (hPa); *O*_10_: oxygen value representing 10% of the range between the maximum and minimum values, i.e., when 90% of the total oxygen (hPa) has been consumed; *t_O_min_*: time in hours it took to consume all the oxygen, i.e., ∆*O_max_min_* (h); *t*_*O*_90_: time it took to reach *O*_90_ (h); *t*_*O*_10_: time to reach *O*_10_ (h); *t_O_int_*: time to fall to 50% air saturation; *AO_max_min_*: area under the oxygen consumption curve between maximum and minimum oxygen (hPa); *A_max_min_*: area under the oxygen consumption curve (hPa). Subsequently, the 3 replicates were combined to obtain a representative average curve for each wine (mean ± SD).

### 3.7. Statistical Analysis

Statistical analysis was performed with the statistical program Statgraphics Centurion (version 19 X-64 StatPoint, Inc., Warrenton, VA, USA), performing an ANOVA according to Tukey’s test (*p* < 0.05) and an analysis of correlations with Pearson’s coefficient.

## 4. Conclusions

The SpectrO_2_ device allows the simultaneous measurement of oxygen and the visible spectrum with precision, accuracy, and repeatability, as well as under watertight conditions, for the measurement of the kinetics of dissolved oxygen consumption of the samples. The SpectrO_2_ offers the possibility to monitor the kinetics of oxygen consumption simultaneously with a spectral scan at each moment of the kinetics without altering the optoluminescent measurement of oxygen. The results obtained with the monitoring of commercial wines show information on the changes that occurred in the visible spectrum during the whole oxygen consumption process, thus being a tool for the analysis of spectral changes during oxygen consumption, which is novel.

Future research will focus on evaluating the effect of specific amounts of oxygen in certain regions of the ultraviolet visible spectrum, both in white and red wines, to determine the influence of grape variety, origin, winery treatments, bottling, corking, etc., and thus allowing us to know the response of the wines to specific amounts of oxygen.

This advanced instrument is capable of assessing dissolved oxygen levels and the visible spectrum in a single measurement, which is an advantageous and powerful method for the wine industry.

## Figures and Tables

**Table 1:** Consumption kinetics parameters of commercial wines analyzed by SpectroO_2_.

Wine	*O_max_*	*O_min_*	∆*O_max_min_*	*O_int_*	*O*_90_	∆*O*_90_10_	*O*_10_	*t_O_min_*	*t*_*O*_90_	*t*_*O*_10_	*t_O_int_*	*AO_max_min_*	*A_max_min_*
Red wine 1 (*n* = 9)	168.7 ± 1.9 b	63.0 ± 1.8 a	105.7 ± 2.2 d	115.9 ± 1.5 a	157.6 ± 1.9 d	84.0 ± 1.9 d	73.7 ± 1.7 a	147.2 ± 3.1 a	4.6 ± 0.7 c	86.1 ± 6.5 a	29.0 ± 1.4 ab	4051 ± 143 bc	13,468 ± 360 a
Red wine 2 (*n* = 3)	167.2 ± 1.2 ab	85.9 ± 8.8 b	81.3 ± 7.7 c	126.5 ± 5.0 b	158.3 ± 6.5 c	64.1 ± 6.5 c	94.2 ± 8.1 b	135.7 ± 11.8 a	1.8 ± 0.4 ab	104.3 ± 22.3 ab	22.0 ± 11.3 a	3215 ± 1416 b	15,476 ± 286 b
Red wine 3 (*n* = 3)	169.5 ± 4.7 b	61.5 ± 5.8 a	108.1 ± 4.7 d	115.5 ± 4.7 a	157.8 ± 3.8 d	85.3 ± 3.8 d	72.5 ± 5.7 a	205.4 ± 4.5 b	2.3 ± 0.9 ab	132.5 ± 60.5 bc	25.9 ± 3.4 ab	5039 ± 1480 c	17,841 ± 281 c
White wine 1 (*n* = 3)	167.8 ± 0.8 ab	129.9 ± 4.9 d	37.9 ± 4.4 a	148.8 ± 2.7 d	163.9 ± 3.4 a	30.2 ± 3.4 a	133.7 ± 4.4 d	193.2 ± 1.5 b	3.8 ± 1.5 bc	114.0 ± 4.1 abc	33.4 ± 2.2 b	1795 ± 296 a	27,090 ± 656 d
White wine 2 (*n* = 3)	165.0 ± 1.2 a	102.8 ± 5.6 c	62.3 ± 5.8 b	133.9 ± 2.8 c	158.6 ± 4.6 b	49.3 ± 4.6 b	109.4 ± 4.8 c	205.3 ± 29.4 b	2.6 ± 0.8 ab	151.7 ± 13.8 c	28.7 ± 4.7 b	3270 ± 623 b	27,884 ± 1001 d

*O_max_*: maximum/initial oxygen value (hPa); *O_min_*: minimum/residual oxygen value (hPa); ∆*O_max_min_*: total oxygen consumed (hPa); *O_int_*: oxygen value when half of the total consumption time has elapsed (hPa); *O*_90_: oxygen representing 90% of the range between the maximum and minimum values, i.e., when the first 10% of total oxygen (hPa) had been consumed; ∆*O*_90_10_: variation between 90% and 10% of oxygen (hPa); *O*_10_: oxygen value that represents 10% of the range between the maximum and minimum values (hPa); *t_O_min_*: time in hours that it took to consume all the oxygen (h); *t*_*O*_90_: time when *O*_90_ is reached (h); *t*_*O*_10_: time when *O*_10_ is reached (h); *t_O_int_*: time to 50% air saturation (h); *AO_max_min_*: area under the oxygen consumption curve between maximum and minimum oxygen (hPa/h); *A_max_min_*: area under the oxygen consumption curve (hPa/h). For each parameter, different letters indicate significant differences between wines.

**Table 2:** Change in wine absorbance with respect to the initial absorbance in each oxygen consumption section; the three highest absorbance losses are highlighted in red, and the three highest absorbance increases are highlighted in green.

	Red Wine 1						Red Wine 2						Red Wine 3							White Wine 1						White Wine 2						
nm	0–10 h	0–20 h	0–30 h	0–50 h	0–75 h	0–147 h	0–10 h	0–20 h	0–30 h	0–50 h	0–75 h	0–136 h	0–10 h	0–20 h	0–30 h	0–50 h	0–75 h	0–150 h	0–205 h	0–10 h	0–20 h	0–30 h	0–50 h	0–75 h	0–193 h	0–10 h	0–20 h	0–30 h	0–50 h	0–75 h	0–150 h	0–205 h
390	−0.3%	−0.2%	−1.4%	0.5%	0.3%	0.8%	−0.5%	−0.2%	0.5%	1.1%	1.9%	6.1%	−0.6%	−0.6%	−0.5%	1.6%	1.4%	3.6%	5.1%	−5.6%	−4.7%	−3.1%	−2.6%	−1.3%	10.0%	−0.1%	−3.0%	−0.6%	0.6%	2.3%	12.9%	15.7%
400	−1.8%	−1.2%	−2.2%	−0.7%	−0.3%	0.1%	−0.5%	0.0%	0.4%	1.7%	2.4%	8.2%	−0.9%	−0.4%	−0.2%	2.1%	2.2%	4.2%	6.9%	−8.1%	−7.0%	−5.7%	−5.2%	−3.2%	9.4%	0.2%	−3.2%	−0.5%	0.2%	2.7%	15.2%	18.0%
410	0.0%	−0.7%	−1.2%	0.6%	0.8%	1.6%	−0.7%	0.0%	0.9%	1.9%	3.0%	9.5%	0.0%	1.4%	2.0%	4.1%	4.3%	6.9%	9.5%	−9.9%	−7.7%	−6.4%	−6.3%	−4.9%	10.3%	0.5%	−4.0%	−0.2%	0.7%	3.4%	16.9%	20.4%
420	0.2%	1.1%	−0.1%	1.4%	1.8%	2.9%	0.9%	−0.7%	0.8%	1.9%	3.0%	10.4%	−0.2%	−0.3%	1.0%	3.0%	3.4%	6.1%	8.6%	−11.4%	−8.9%	−7.6%	−7.2%	−5.5%	9.5%	0.0%	−3.9%	−0.2%	0.0%	3.0%	16.7%	20.3%
430	0.5%	0.6%	−0.1%	1.3%	1.5%	2.6%	−1.6%	−1.3%	0.0%	1.1%	2.1%	9.7%	−1.6%	−0.9%	−0.2%	2.1%	2.2%	5.4%	7.4%	−13.3%	−11.6%	−9.6%	−9.6%	−7.9%	7.9%	−0.5%	−4.0%	−0.4%	0.5%	2.5%	17.5%	21.5%
440	−0.9%	−0.4%	−1.1%	0.5%	0.4%	2.0%	−2.2%	−1.7%	−0.5%	0.6%	1.7%	9.5%	−2.8%	−1.8%	−1.0%	1.0%	1.6%	4.6%	7.6%	−15.6%	−14.7%	−12.4%	−12.5%	−10.5%	6.0%	−0.4%	−3.1%	0.3%	0.2%	2.7%	17.4%	21.6%
450	−1.9%	−1.8%	−3.0%	−1.7%	−1.2%	−0.3%	−1.0%	−0.8%	0.3%	1.4%	2.3%	10.8%	−0.7%	0.0%	0.7%	3.1%	3.0%	6.6%	9.4%	−10.5%	−0.8%	1.4%	2.5%	3.0%	19.4%	0.6%	−1.7%	2.4%	1.3%	4.0%	16.2%	21.0%
460	−1.5%	−1.5%	−2.1%	−0.8%	−0.7%	0.3%	−0.3%	0.1%	0.8%	1.9%	3.2%	10.2%	−0.2%	0.8%	1.2%	3.6%	4.2%	7.4%	10.0%	−6.8%	−7.0%	−5.5%	−5.5%	−4.7%	4.6%	0.8%	−0.4%	2.2%	2.7%	4.3%	12.1%	15.1%
470	−1.7%	−1.1%	−1.8%	−1.0%	−0.6%	0.3%	−0.1%	0.2%	1.1%	2.1%	3.3%	10.0%	−0.1%	0.6%	1.5%	3.2%	4.0%	6.7%	9.3%	−3.1%	−2.7%	−2.5%	−2.2%	−0.9%	3.9%	0.3%	1.0%	2.8%	3.3%	4.5%	8.6%	11.5%
480	−1.4%	−1.0%	−1.6%	−1.1%	−1.1%	−0.3%	0.0%	0.3%	1.1%	1.9%	2.9%	9.1%	0.4%	1.2%	1.6%	3.0%	3.8%	6.4%	8.8%	−1.7%	−0.9%	−0.7%	−0.5%	0.2%	2.5%	1.2%	2.1%	3.1%	4.5%	5.4%	7.1%	8.7%
490	−3.0%	−2.8%	−3.4%	−3.0%	−3.1%	−3.2%	−0.7%	−0.3%	0.2%	0.8%	1.5%	6.4%	−0.5%	0.1%	0.5%	1.8%	2.5%	4.7%	6.3%	−1.5%	−1.3%	−1.2%	−0.2%	0.3%	2.1%	0.8%	1.9%	2.9%	4.0%	4.8%	6.7%	8.2%
500	−1.4%	−1.3%	−2.0%	−1.9%	−2.2%	−2.8%	−0.4%	−0.3%	0.1%	0.6%	1.1%	5.0%	−0.1%	0.2%	0.7%	1.6%	2.1%	3.7%	5.2%	−0.6%	−0.4%	0.3%	0.3%	1.4%	3.0%	1.3%	2.5%	3.4%	4.6%	5.3%	7.2%	8.4%
510	−0.8%	−1.0%	−1.8%	−1.9%	−2.5%	−3.6%	−0.4%	−0.2%	0.0%	0.3%	0.7%	3.7%	−0.1%	0.3%	0.5%	1.2%	1.3%	2.4%	3.4%	−0.1%	0.6%	0.6%	1.2%	1.6%	3.4%	1.6%	2.6%	3.6%	4.6%	5.3%	6.9%	7.9%
520	−1.0%	−1.2%	−1.8%	−2.3%	−3.1%	−4.7%	−0.4%	−0.3%	−0.2%	−0.1%	0.1%	2.2%	−0.1%	−0.1%	0.1%	0.5%	0.5%	1.1%	1.8%	−0.2%	0.1%	0.3%	0.7%	1.3%	2.8%	1.1%	1.8%	2.9%	4.1%	4.7%	5.9%	7.1%
530	−1.2%	−1.4%	−2.3%	−2.9%	−3.9%	−5.9%	−0.7%	−0.7%	−0.6%	−0.6%	−0.5%	0.6%	−0.3%	−0.4%	−0.4%	−0.2%	−0.2%	−0.1%	0.2%	−0.1%	0.1%	0.4%	0.8%	1.2%	2.2%	0.5%	1.3%	2.4%	3.4%	4.0%	4.9%	6.0%
540	0.0%	−0.4%	−1.3%	−2.1%	−3.2%	−5.6%	−1.9%	−2.0%	−1.9%	−2.1%	−2.2%	−1.6%	−1.7%	−1.8%	−1.9%	−1.8%	−2.1%	−2.3%	−2.3%	−1.2%	−1.0%	−0.8%	−0.4%	0.1%	0.7%	−0.7%	0.2%	1.1%	1.8%	2.4%	3.3%	4.1%
550	−0.1%	−0.4%	−1.4%	−2.3%	−3.5%	−5.9%	−1.6%	−1.7%	−1.8%	−2.0%	−2.1%	−1.7%	−1.4%	−1.6%	−1.8%	−1.9%	−2.2%	−2.7%	−3.0%	−0.2%	0.3%	0.2%	0.6%	0.9%	1.5%	−0.7%	0.2%	0.8%	1.7%	2.0%	2.9%	3.6%
560	−1.0%	−1.4%	−2.3%	−3.2%	−4.4%	−6.9%	−1.0%	−1.0%	−1.1%	−1.3%	−1.3%	−0.5%	−0.6%	−0.9%	−1.1%	−1.4%	−1.8%	−2.6%	−3.2%	−0.2%	0.2%	0.0%	0.5%	0.8%	1.6%	−0.3%	0.6%	1.4%	1.9%	2.5%	3.2%	3.9%
570	−0.9%	−1.3%	−2.2%	−3.1%	−4.3%	−6.5%	−0.8%	−0.9%	−0.8%	−1.0%	−0.9%	0.7%	−0.6%	−0.9%	−1.2%	−1.6%	−2.1%	−3.1%	−3.7%	−0.5%	−0.3%	−0.4%	0.2%	0.2%	1.1%	−0.4%	0.5%	1.0%	1.5%	1.9%	2.7%	3.4%
580	−1.1%	−1.5%	−2.3%	−3.2%	−4.3%	−6.4%	−0.8%	−0.7%	−0.7%	−0.7%	−0.5%	2.2%	−0.8%	−1.1%	−1.4%	−1.8%	−2.4%	−3.4%	−4.1%	−1.1%	−0.9%	−0.7%	−0.4%	−0.1%	0.7%	−0.8%	0.1%	0.7%	1.3%	1.8%	2.6%	3.3%
590	−1.0%	−1.3%	−2.0%	−2.9%	−3.8%	−5.5%	−0.5%	−0.4%	−0.2%	−0.1%	0.3%	4.3%	−0.7%	−1.0%	−1.3%	−1.7%	−2.2%	−3.1%	−3.7%	−0.9%	−0.7%	−0.7%	−0.5%	−0.2%	0.9%	−0.6%	0.0%	0.7%	1.2%	1.5%	2.4%	3.4%
600	−0.6%	−0.9%	−1.5%	−2.1%	−3.0%	−4.3%	−0.1%	0.2%	0.5%	0.6%	1.2%	6.4%	−0.4%	−0.7%	−0.9%	−1.2%	−1.5%	−2.2%	−2.5%	−0.8%	−0.8%	−0.6%	−0.3%	0.3%	0.5%	−0.6%	0.1%	0.5%	1.0%	1.4%	2.4%	3.2%
610	−1.1%	−1.2%	−1.7%	−2.2%	−2.8%	−3.8%	0.1%	0.4%	0.8%	1.0%	1.7%	8.0%	−0.5%	−0.6%	−0.7%	−0.8%	−1.0%	−1.2%	−1.0%	−2.0%	−2.0%	−2.3%	−2.0%	−1.6%	−1.2%	−0.9%	−0.4%	0.2%	0.7%	0.9%	1.9%	2.9%
620	−0.3%	−0.3%	−0.6%	−0.9%	−1.4%	−1.9%	0.9%	1.4%	1.8%	2.1%	2.9%	9.9%	0.1%	0.1%	0.2%	0.3%	0.3%	0.6%	1.3%	−0.6%	−0.6%	−0.5%	−0.1%	0.2%	0.6%	−0.9%	−0.1%	0.4%	0.9%	1.3%	2.0%	2.8%
630	0.0%	0.1%	−0.1%	−0.2%	−0.4%	−0.6%	1.5%	2.1%	2.7%	3.0%	3.8%	11.7%	0.5%	0.7%	0.9%	1.2%	1.5%	2.3%	3.5%	−0.8%	−0.5%	−0.8%	−0.4%	−0.1%	0.5%	−0.6%	0.1%	0.3%	0.9%	1.3%	2.0%	2.6%
640	0.2%	0.5%	0.5%	0.6%	0.6%	0.9%	2.1%	2.9%	3.5%	3.7%	4.5%	12.9%	0.8%	1.2%	1.5%	2.0%	2.5%	3.8%	5.7%	−0.1%	−0.1%	−0.1%	0.2%	0.3%	1.0%	−0.5%	0.2%	0.6%	1.3%	1.6%	2.1%	2.8%
650	0.3%	0.8%	0.9%	1.3%	1.5%	2.2%	2.6%	3.5%	4.1%	4.3%	5.0%	13.7%	1.1%	1.4%	1.9%	2.6%	3.2%	5.0%	7.5%	−0.3%	−0.1%	0.0%	0.3%	0.5%	1.0%	−0.2%	0.6%	0.9%	1.5%	1.6%	2.2%	3.0%
660	0.3%	1.0%	1.2%	1.8%	2.1%	3.2%	3.1%	4.1%	4.6%	4.7%	5.3%	13.9%	1.3%	1.8%	2.3%	3.0%	3.9%	6.1%	8.9%	−0.2%	−0.2%	0.0%	0.5%	0.6%	0.9%	−0.4%	0.4%	0.7%	1.4%	1.5%	2.4%	2.8%
670	0.3%	1.1%	1.3%	2.0%	2.5%	3.9%	3.7%	4.6%	5.1%	4.9%	5.6%	13.8%	1.7%	2.2%	2.6%	3.4%	4.4%	6.7%	10.0%	−0.2%	−0.3%	0.1%	0.4%	0.6%	1.2%	−0.7%	0.1%	0.3%	0.8%	1.3%	2.1%	2.6%
680	0.4%	1.2%	1.4%	2.3%	2.7%	4.3%	3.8%	4.8%	5.1%	4.7%	5.2%	13.3%	1.7%	2.2%	2.6%	3.4%	4.5%	7.0%	10.4%	0.0%	0.2%	−0.3%	0.0%	0.5%	0.8%	−0.5%	0.0%	0.7%	1.1%	1.4%	2.2%	3.0%
690	0.2%	1.0%	1.2%	2.1%	2.8%	4.4%	4.2%	5.1%	5.4%	5.1%	5.4%	12.8%	1.9%	2.5%	2.9%	3.8%	4.8%	7.4%	10.7%	−0.2%	−0.3%	−0.6%	−0.3%	0.3%	0.6%	−0.4%	0.3%	0.5%	1.1%	1.3%	2.1%	2.4%
700	0.3%	1.3%	1.4%	2.3%	2.9%	4.5%	3.5%	4.5%	4.8%	4.2%	4.6%	11.8%	1.5%	2.1%	2.5%	3.4%	4.4%	6.9%	10.5%	−0.3%	−0.1%	0.1%	0.6%	0.6%	0.9%	−0.2%	0.4%	0.6%	1.1%	1.3%	1.9%	2.5%
710	−0.4%	0.6%	0.6%	1.5%	2.2%	3.7%	4.2%	4.7%	4.9%	4.2%	4.4%	11.2%	2.1%	2.4%	2.7%	3.6%	4.5%	7.1%	10.4%	0.3%	0.3%	−0.5%	0.0%	0.4%	0.5%	−0.8%	0.1%	0.3%	0.6%	0.8%	1.3%	1.9%
720	−0.5%	0.2%	0.4%	1.3%	1.7%	3.2%	3.7%	4.4%	4.3%	3.4%	3.6%	9.8%	1.7%	2.0%	2.3%	3.0%	4.0%	6.4%	9.8%	−0.3%	−0.4%	−0.5%	−0.2%	0.0%	0.0%	−0.4%	0.3%	0.6%	1.3%	1.4%	1.7%	2.1%
730	−0.7%	0.0%	0.1%	1.2%	1.6%	2.9%	3.4%	4.0%	4.1%	3.1%	3.2%	9.5%	1.5%	1.8%	2.1%	2.9%	3.9%	6.3%	9.8%	−0.4%	−0.5%	−0.3%	−0.1%	0.2%	0.7%	0.2%	0.9%	0.9%	1.4%	1.5%	1.9%	2.4%
740	−0.8%	−0.2%	−0.3%	0.6%	0.9%	2.2%	3.7%	4.2%	4.2%	3.1%	3.1%	9.0%	1.9%	2.2%	2.4%	3.1%	4.1%	6.4%	9.7%	−0.7%	−0.9%	−0.6%	−0.5%	−0.4%	−0.4%	0.1%	0.7%	0.5%	1.1%	1.3%	1.7%	2.5%
750	−1.1%	−0.3%	−0.4%	0.8%	1.1%	1.6%	3.2%	3.8%	3.6%	2.2%	2.3%	8.0%	1.5%	1.9%	2.1%	2.4%	3.5%	5.8%	9.0%	−0.3%	−0.7%	−0.7%	−0.3%	−0.1%	−0.2%	0.2%	0.6%	0.7%	1.2%	1.1%	1.8%	2.3%
760	−1.1%	−0.6%	−0.9%	0.1%	0.3%	1.3%	3.1%	3.6%	3.4%	2.2%	2.3%	7.2%	1.5%	1.8%	2.0%	2.8%	3.7%	5.8%	8.7%	−0.3%	−0.3%	−0.6%	−0.3%	0.2%	−0.3%	0.3%	0.6%	0.9%	1.3%	1.6%	2.4%	2.5%
770	−0.9%	−0.3%	−0.6%	0.1%	0.5%	1.0%	2.7%	3.1%	2.9%	1.8%	1.6%	6.5%	1.3%	1.4%	1.7%	2.4%	3.2%	5.3%	8.2%	−0.4%	−0.4%	−0.5%	−0.4%	−0.5%	−0.4%	0.4%	1.0%	0.9%	1.4%	1.6%	2.1%	2.5%
780	−0.9%	−0.3%	−0.3%	0.1%	0.4%	0.9%	2.7%	3.3%	2.9%	1.5%	1.5%	6.2%	1.3%	1.7%	1.6%	2.3%	3.3%	5.2%	8.2%	−0.6%	−0.6%	−1.2%	−1.1%	−0.8%	−0.7%	−0.1%	0.3%	0.3%	0.8%	1.1%	1.7%	2.4%
790	−1.5%	−1.1%	−1.0%	−0.4%	−0.3%	0.1%	2.8%	3.1%	3.0%	1.4%	1.2%	6.2%	1.4%	1.6%	1.6%	2.1%	3.0%	4.9%	8.0%	−0.4%	−0.3%	−0.6%	−0.4%	−0.1%	0.1%	−0.7%	−0.5%	−0.1%	0.4%	0.5%	1.1%	1.8%
800	−1.6%	−0.9%	−1.2%	−0.6%	−0.6%	0.0%	2.5%	2.6%	2.4%	0.9%	0.7%	5.8%	0.9%	1.0%	1.1%	1.6%	2.3%	4.2%	7.4%	−0.3%	−0.6%	−1.4%	−1.3%	−0.9%	−0.1%	−0.5%	−0.1%	0.3%	0.4%	0.7%	0.7%	1.2%

**Table 3:** Correlation coefficient between consumed oxygen and absorbance at different wavelengths during oxygen consumption by different wines; significant correlations are highlighted in bold, the 10 most significant negative correlations in red, and the 10 most significant positive correlations in green.

λ (nm)	Red Wine 1	Red Wine 2	Red Wine 3	White Wine 1	White Wine 2	λ (nm)	Red Wine 1	Red Wine 2	Red Wine 3	White Wine 1	White Wine 2
390	0.1629	0.5716	**0.759**	0.7064	**0.8334**	600	**−0.9561**	0.5201	**−0.972**	0.7022	**0.9199**
395	**0.8428**	0.5678	**0.827**	0.6826	**0.8469**	605	**−0.9505**	0.5495	**−0.978**	0.6883	**0.9519**
400	**0.7816**	0.6008	**0.955**	0.6431	**0.8500**	610	**−0.9853**	0.5276	**−0.948**	−0.1849	**0.8345**
405	**0.8625**	0.4467	**0.849**	0.6030	**0.8323**	615	**−0.9736**	0.5885	**0.059**	0.3676	**0.8862**
410	0.5082	0.6128	**0.891**	0.5993	**0.8353**	620	**−0.9375**	0.6218	**0.818**	0.6377	**0.9266**
415	0.3637	0.5760	**0.878**	0.5902	**0.8324**	625	**−0.9247**	0.6374	**0.882**	0.8301	**0.8929**
420	0.5120	0.5770	**0.921**	0.6042	**0.8405**	630	**−0.8603**	0.6585	**0.900**	0.6131	**0.9185**
425	0.2329	0.5553	**0.891**	0.5951	**0.8431**	635	**0.4612**	0.6773	**0.913**	**0.7496**	**0.9273**
430	0.1923	0.5239	**0.862**	0.5430	**0.8494**	640	**0.9497**	0.6948	**0.920**	**0.7543**	**0.9377**
435	**0.7628**	0.3779	**0.570**	0.3465	**0.7951**	645	**0.9760**	0.6930	**0.913**	**0.8647**	**0.9200**
440	0.6096	0.5396	**0.861**	0.5110	**0.8544**	650	**0.9587**	0.7084	**0.909**	**0.7246**	**0.9549**
445	0.0135	0.5015	**0.868**	0.5216	**0.8636**	655	**0.9677**	0.7156	**0.906**	**0.8001**	**0.9387**
450	−0.0714	0.5259	**0.854**	0.5364	**0.8711**	660	**0.9642**	0.7325	**0.915**	**0.7444**	**0.9390**
455	0.1190	0.5816	**0.904**	0.4380	**0.8826**	665	**0.9592**	0.7440	**0.914**	**0.9012**	**0.9102**
460	−0.6625	0.5733	**0.884**	**0.9105**	**0.9055**	670	**0.9555**	0.7370	**0.907**	**0.8210**	**0.9365**
465	0.2303	0.6031	**0.910**	**0.9236**	**0.9266**	675	**0.9467**	0.7476	**0.912**	**0.8981**	**0.9110**
470	0.1104	0.6050	**0.901**	0.6431	**0.9413**	680	**0.9555**	0.7418	**0.907**	**0.7330**	**0.9336**
475	0.4174	0.6168	**0.921**	0.6897	**0.9576**	685	**0.9357**	0.7537	**0.907**	**0.7201**	**0.9567**
480	−0.0579	0.6181	**0.917**	**0.7997**	**0.9874**	690	**0.9420**	**0.7815**	**0.927**	0.4937	**0.9486**
485	−0.6642	0.5567	**0.875**	0.6929	**0.9822**	695	**0.9355**	0.7522	**0.906**	**0.7669**	**0.9732**
490	−0.7202	0.5403	**0.881**	**0.7814**	**0.9781**	700	**0.9316**	0.7422	**0.904**	**0.8902**	**0.9468**
495	**−0.8686**	0.5302	**0.893**	**0.7388**	**0.9923**	705	**0.9284**	0.7476	**0.911**	**0.8001**	**0.8358**
500	**−0.9729**	0.5261	**0.894**	**0.8390**	**0.9883**	710	**0.8846**	0.7343	**0.903**	0.5769	**0.8195**
505	**−0.9808**	0.5208	**0.892**	**0.8940**	**0.9893**	715	**0.8923**	0.7364	**0.907**	0.4309	**0.9286**
510	**−0.9770**	0.5069	**0.889**	**0.8986**	**0.9892**	720	**0.8473**	0.7041	**0.887**	0.5509	**0.9135**
515	**−0.9595**	0.4641	**0.904**	**0.9184**	**0.9876**	725	**0.8779**	0.7036	**0.897**	−0.0467	**0.9644**
520	**−0.9536**	0.3988	**0.837**	**0.8758**	**0.9875**	730	**0.8405**	0.6763	**0.887**	**0.8210**	**0.9632**
525	**−0.9541**	0.2800	**0.760**	**0.8837**	**0.9795**	735	**0.8232**	0.6942	**0.894**	0.6751	**0.8542**
530	**−0.9573**	0.0514	**0.370**	**0.9119**	**0.9828**	740	**0.7450**	0.6929	**0.894**	0.2369	**0.9319**
535	**−0.9725**	−0.5096	**−0.839**	**0.8518**	**0.9751**	745	**0.7563**	0.6650	**0.888**	−0.4675	**0.9156**
540	**−0.8912**	**−0.8112**	**−0.853**	0.1640	**0.9364**	750	**0.7684**	0.6645	**0.892**	0.4518	**0.9463**
545	0.4292	**−0.8718**	**−0.876**	−0.2214	**0.8365**	755	0.7100	0.5949	**0.869**	0.2467	**0.9679**
550	**−0.9791**	**−0.8907**	**−0.979**	0.5491	**0.9579**	760	0.5872	0.6635	**0.905**	0.4134	**0.9943**
555	**−0.9588**	**−0.8443**	**−0.966**	**0.7447**	**0.9450**	765	0.5850	0.6597	**0.915**	−0.2791	**0.9550**
560	**−0.9466**	−0.6750	**−0.965**	**0.8990**	**0.9645**	770	0.6833	0.5309	**0.851**	−0.3695	**0.9695**
565	**−0.9471**	−0.4338	**−0.956**	**0.8314**	**0.9611**	775	0.4832	0.6422	**0.899**	0.3983	**0.9546**
570	**−0.9494**	−0.0767	**−0.959**	**0.8084**	**0.9475**	780	0.8649	0.5883	**0.886**	−0.8028	**0.8796**
575	**−0.9551**	0.0982	**−0.962**	0.6888	**0.9112**	785	0.3836	0.6006	**0.859**	0.2559	**0.9112**
580	**−0.9600**	0.2341	**−0.963**	0.6291	**0.9254**	790	0.4205	0.5057	**0.866**	0.5779	**0.8166**
585	**−0.9664**	0.3463	**−0.968**	0.6232	**0.9296**	795	0.3400	0.4824	**0.859**	−0.1861	**0.8161**
590	**−0.9623**	0.4133	**−0.966**	0.7037	**0.9169**	800	0.1590	0.5360	**0.845**	0.1647	**0.8140**
595	**−0.9590**	0.4721	**−0.969**	0.6613	**0.9338**						

**Table 4:** Oenological parameters of commercial wines.

Wine	Red Wine 1	Red Wine 2	Red Wine 3	White Wine 1	White Wine 2
Variety	Tinta de Toro	Mandón	Cabernet Sauvignon	Sauvignon blanc	Verdejo
Vintage	2021	2021	2016	2021	2019
Type *	-	-	6	-	+
pH	3.60 ± 0.0	3.30 ± 0.0	3.25 ± 0.0	3.10 ± 0.0	3.24 ± 0.0
TA (g/L)	5.4 ± 0.1	5.0 ± 0.0	4.8 ± 0.0	5.4 ± 0.0	5.3 ± 0.0
CI	13.62 ± 0.02	7.69 ± 0.01	11.25 ± 0.03	0.33 ± 0.01	0.63 ± 0.02
%420	36	34	41	100	100
%520	51	52	46	-	-
%620	13	14	13	-	-
AS (%vol)	14.51 ± 0.01	10.51 ± 0.01	13.51 ± 0.01	13.01 ± 0.01	13.51 ± 0.01
F–SO_2_ (mg/L)	1 ± 2.1	1 ± 2.1	1 ± 2.1	17 ± 2.1	1.00 ± 2.1
T–SO_2_ (mg/L)	2 ± 3.5	25 ± 3.5	2 ± 3.5	152 ± 26.1	94.28 ± 3.5
TPI	60.5 ± 0.00	33.9 ± 0.28	65.53 ± 0.11	7.43 ± 0.67	7.43 ± 0.00
ACY (mg/L)	447 ± 11.9	197 ± 5.9	74 ± 6.9	-	-
TP (mg/L)	603 ± 4.2	468 ± 14.3	670 ± 101.5	88 ± 16.4	89 ± 2.1
LPP (mg/L)	397 ± 8.4	82 ± 3.4	373 ± 11.8	3 ± 0.5	6 ± 0.0
HPP (mg/L)	206 ± 4.2	387 ± 10.9	297 ± 89.7	85 ± 16.0	83 ± 2.1

TA: total acidity (g/L tartaric acid); CI: color intensity; AS: alcoholic strength (%vol); FSO_2_: free SO_2_ (mg/L); TSO_2_: total SO_2_ (mg/L); TPI: total phenolic index; ACY: total anthocyanins (mg/L); TP: total phenols (mg/L); LPP: low polymerized phenols (mg/L); HPP: high polymerized phenols (mg/L). * oak wood condition: months of wood contact, - non contact, + fermented in oak barrel.

## Data Availability

The data presented in this study are available on request from the corresponding author. The data are not publicly available due to it will be used for future paper.

## Supplementary Materials

The following supporting information can be downloaded at: https://www.mdpi.com/article/10.3390/molecules29010231/s1, Supplementary Figure S1: Evolution of the wine spectrum during oxygen consumption at 8 different times.
