# Supplementary figures and images for: New System for Simultaneous Measurement of Oxygen Consumption and Changes in Wine Color
Source: Molecules. 2023 Dec 31;29(1):231. doi: 10.3390/molecules29010231 (PMC10780306; doi:10.3390/molecules29010231)

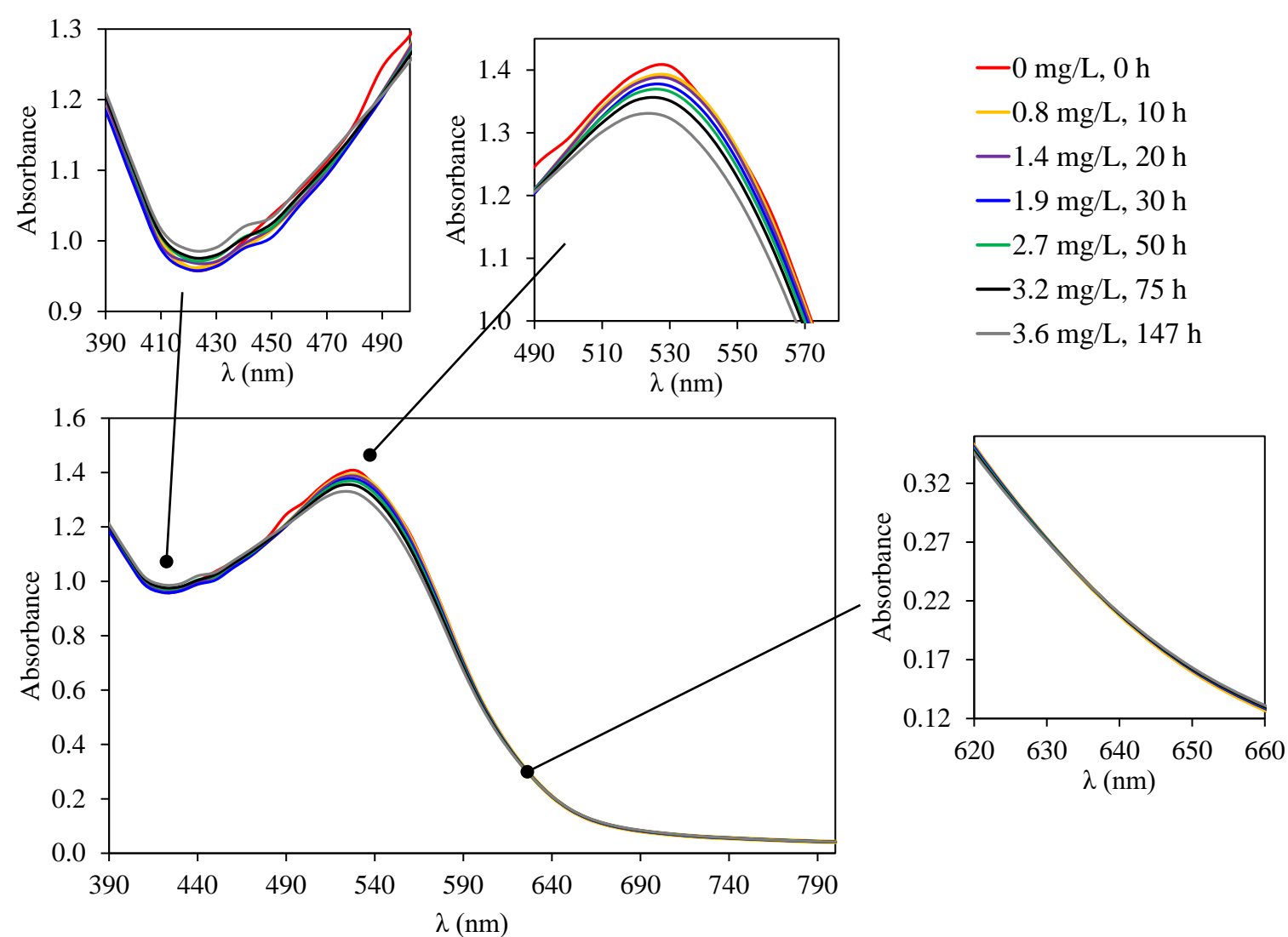

Supplement: Supplementary file 1 [file molecules-29-00231-s001.zip › Figure S1a.pdf]

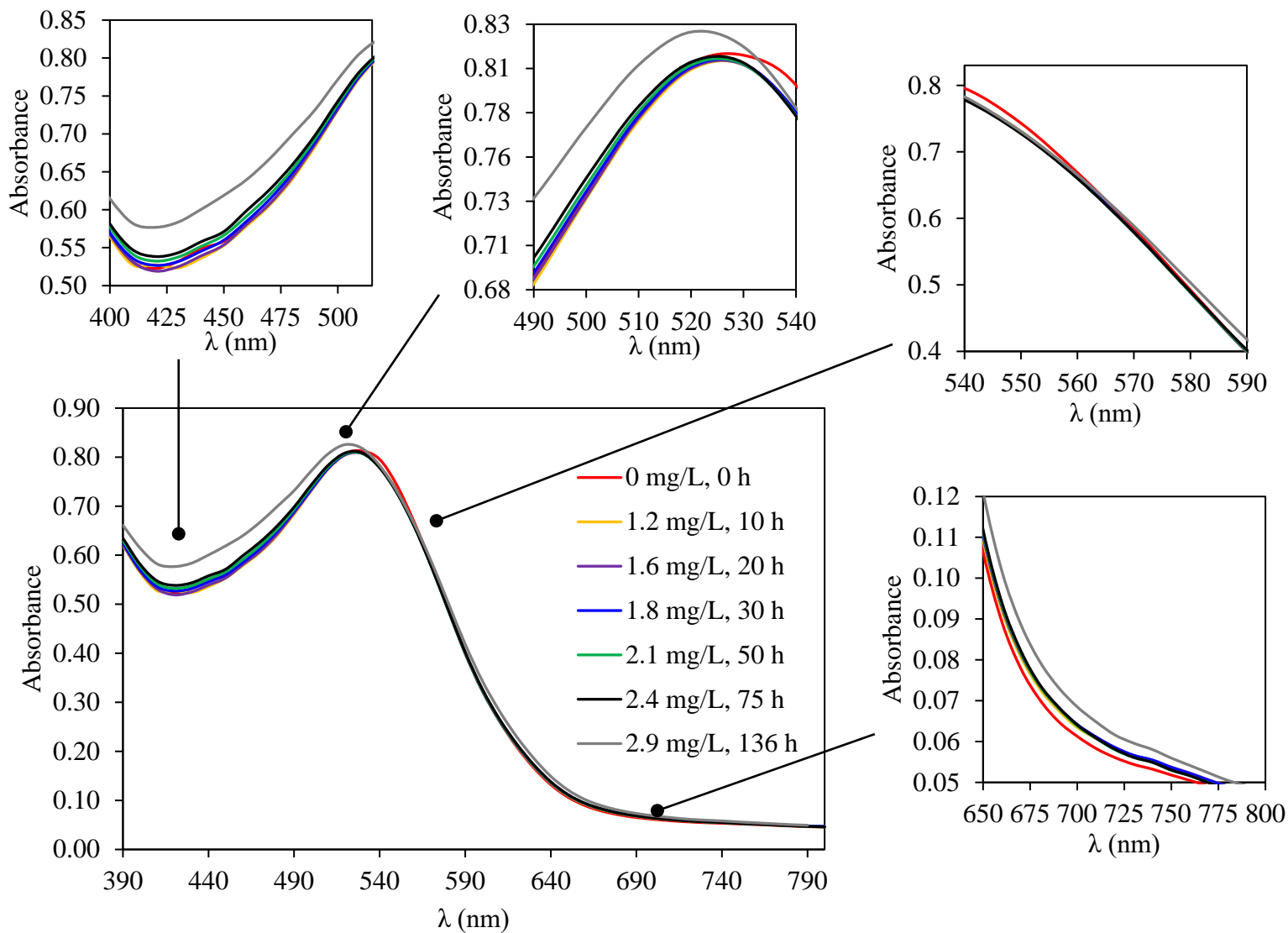

Supplement: Supplementary file 1 [file molecules-29-00231-s001.zip › Figure S1b.pdf]

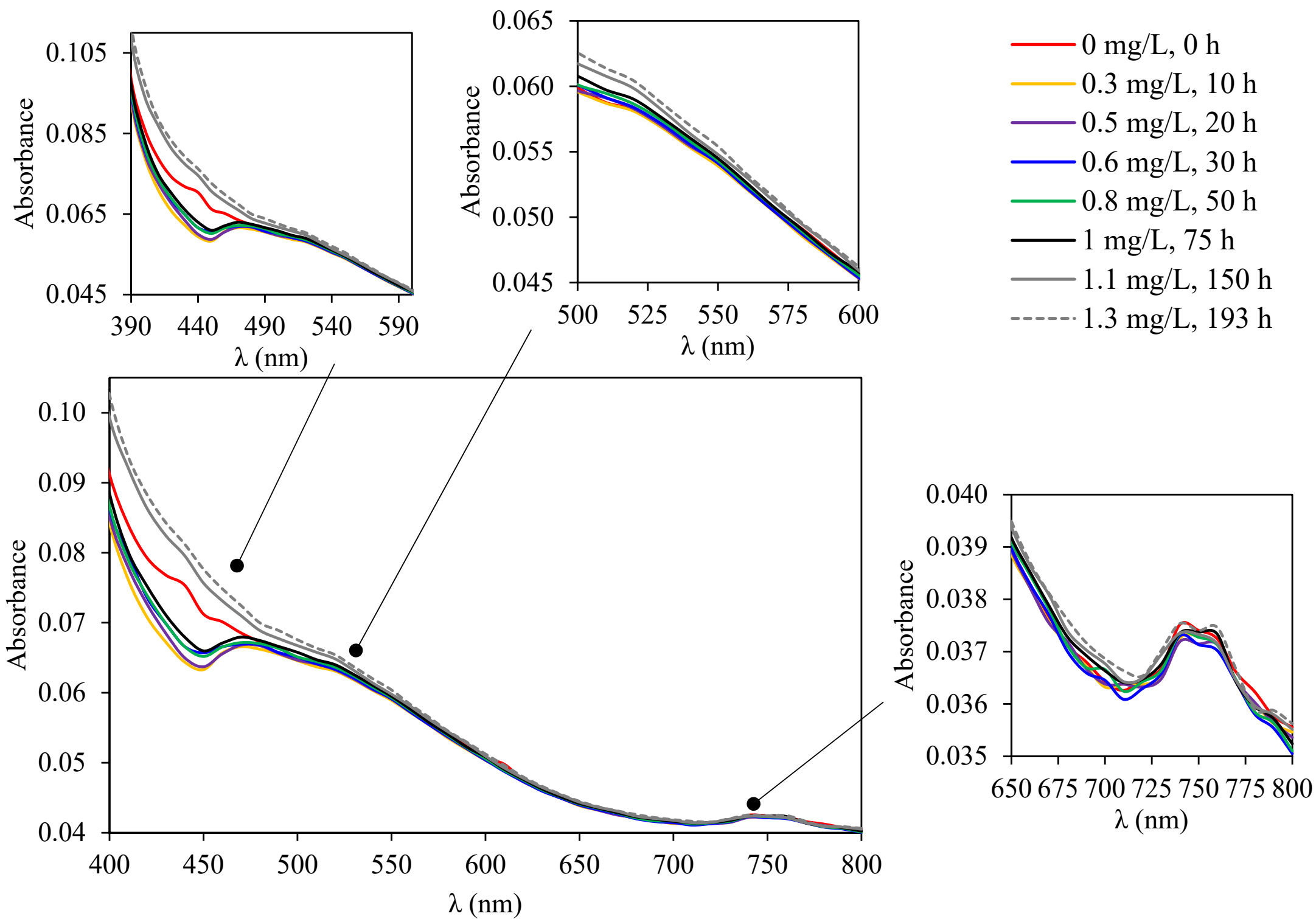

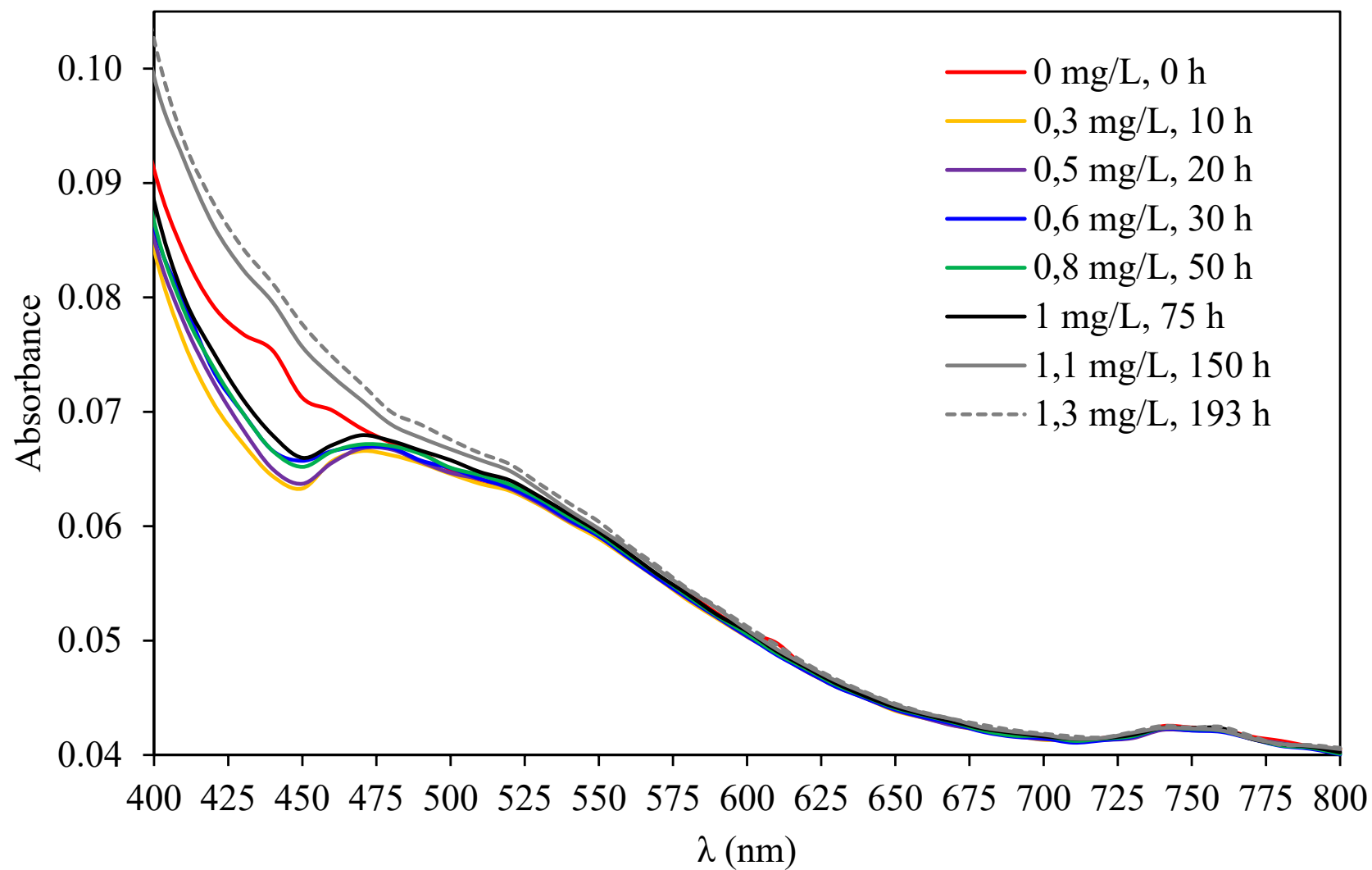

Supplement: Supplementary file 1 [file molecules-29-00231-s001.zip › Figure S1c.pdf]

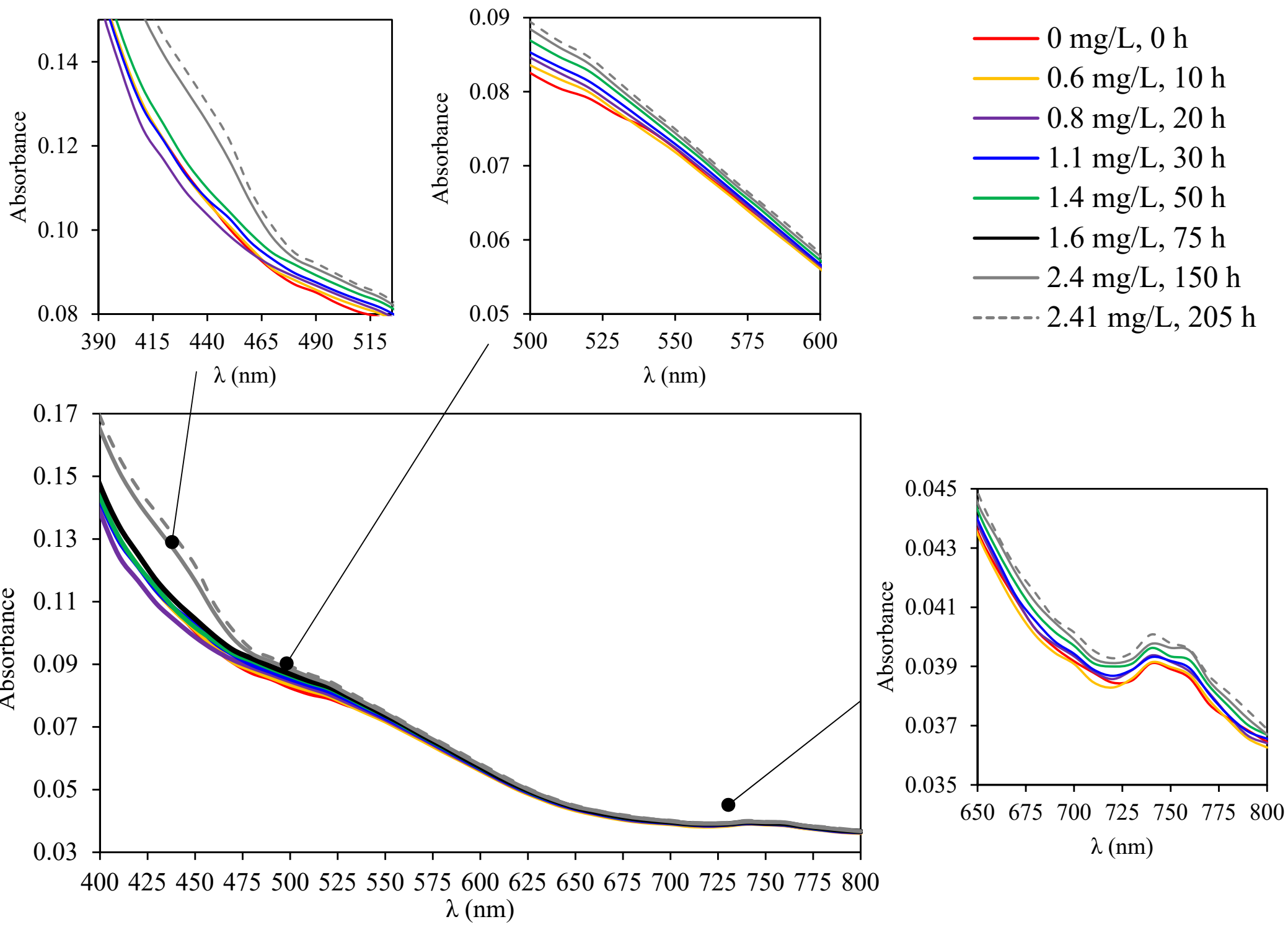

Supplement: Supplementary file 1 [file molecules-29-00231-s001.zip › Figure S1d.pdf]
